# Cope's Rule in a modular organism: Directional evolution without an overarching macroevolutionary trend

**DOI:** 10.1111/evo.13800

**Published:** 2019-07-23

**Authors:** Lee Hsiang Liow, Paul D. Taylor

**Affiliations:** ^1^ Natural History Museum University of Oslo Oslo Norway; ^2^ Department of Biosciences, Centre for Ecological and Evolutionary Synthesis University of Oslo Oslo Norway; ^3^ Natural History Museum London United Kingdom

**Keywords:** Bergmann's Rule, cheilostome bryozoans, fossils, macroevolution, module size

## Abstract

Cope's Rule describes increasing body size in evolutionary lineages through geological time. This pattern has been documented in unitary organisms but does it also apply to module size in colonial organisms? We address this question using 1169 cheilostome bryozoans ranging through the entire 150 million years of their evolutionary history. The temporal pattern evident in cheilostomes as a whole shows no overall change in zooid (module) size. However, individual subclades show size increases: within a genus, younger species often have larger zooids than older species. Analyses of (paleo)latitudinal shifts show that this pattern cannot be explained by latitudinal effects (Bergmann's Rule) coupled with younger species occupying higher latitudes than older species (an “out of the tropics” hypothesis). While it is plausible that size increase was linked to the advantages of large zooids in feeding, competition for trophic resources and living space, other proposed mechanisms for Cope's Rule in unitary organisms are either inapplicable to cheilostome zooid size or cannot be evaluated. Patterns and mechanisms in colonial organisms cannot and should not be extrapolated from the better‐studied unitary organisms. And even if macroevolution simply comprises repeated rounds of microevolution, evolutionary processes occurring within lineages are not always detectable from macroevolutionary patterns.

Body size variations closely reflect important individual differences in life history traits. These include survival, age at sexual maturity and fecundity, as well as physiological traits such as metabolic rate (Peters 1983). Within many groups of metazoans, body size has increased over timescales of millions of years (Payne et al. 2009; Heim et al. 2015), purportedly because of the myriad advantages of being larger. This trend is variously called Cope's Rule or Depéret's Rule and has been explored in groups ranging from ostracodes (Hunt and Roy 2006) to mammals (Alroy 1998), using extinct species from the fossil record (Jablonski 1997), only extant organisms (Baker et al. 2015) or a combination of the two (Bokma et al. 2016).

Many of our insights on life history traits in general, and especially macroevolutionary patterns of changes in size, have come almost entirely from unitary metazoans rather than modular colonial metazoans (Alroy 1998; Hunt and Roy 2006; Novack‐Gottshall and Lanier 2008; Heim et al. 2015). This dearth of insights and theory from clonal or colonial perspectives (Hamilton et al. 1987) is in part because body size, a trait relatively easily measured in unitary organisms, can be difficult to quantify in modular colonial organisms, such as cnidarians, ascidians, and bryozoans, as these often show indeterminate growth (Heino and Kaitala 1999). There is in fact no allometric metabolic constraint on modular iteration (Hughes and Huges 1986), at least in some modular organisms. In addition, modules and entire colonies may be subject to different selective forces and variation at these different levels could have distinguishable impacts on fitness (Tuomi and Vuorisalo 1989; Pedersen and Tuomi 1995; Folse and Roughgarden 2010). There is empirical evidence that module size does contribute importantly to fitness. For instance, module (polyp) size in corals scales in a predictable way, where species with larger polyps exhibit a higher reproductive output per polyp, albeit coupled with a lower reproductive output relative to their investment in somatic tissues (Leuzinger et al. 2003).

But how do module sizes vary through geological time? Do or should module sizes conform to Cope's Rule? The influential paleontologist Norman Newall noted 70 years’ ago that Cope's Rule by no means applied to all groups of organisms and that units of selection may not be limited to the “whole individual” in colonial organisms (Newall 1949). Yet, he also noted that there may be selection for “increasing size in the parent individual or sexual generation” (Newall 1949 p. 109) in colonial organisms. Despite this early interest in the biological implications of module size (see also Elias 1937), little is known about its evolution among colonial organisms.

The aim of this article is, thus, to answer the question: Does Cope's Rule applies to colonial organisms? We chose a group of colonial organisms that have a substantial representation in both the fossil record and in the living marine biota, namely, cheilostome bryozoans. These are the most species‐rich and morphologically disparate clade of extant bryozoans today and thrive in marine habitats globally (Bock and Gordon 2013; Cook et al. 2018a). Using standard models of phenotypic change, we first seek evidence for a general size increase in module (zooid) size among 1169 cheilostome species across the entire 150 million years evolutionary history of the group, representing roughly 8 to 11% of cheilostome species diversity (http://bryozoa.net/diversity.html accessed 6.3.2019 and Horowitz & Pachut 2000. We then examine if the macroevolutionary pattern observed across time is upheld within subclades by answering the question: Do younger species (i.e., putative descendants) tend to have larger zooids than older species (i.e., putative ancestors) within the same genera?

Zooid length varies among cheilostome species by an order of magnitude (Gordon and Taylor 2008; Winston and Vieira 2013). While some of this variation may be due to selection for descendant species with zooid sizes different from ancestral species, ecological factors almost certainly play important roles in directly affecting zooid size. For instance, there is a widely documented tendency for unitary organisms living in higher latitudes to be larger than their counterparts in lower latitudes, in accordance with Bergmann's Rule (see e.g., Meiri and Dayan 2003 and references therein). It has also been shown that zooid sizes of cheilostome species living in higher latitudes tend to be perceptibly larger than their congeners in lower latitudes (Kuklinski and Taylor 2008). How growth forms constrain module size and shape are best studied in plants (Parkhurst and Loucks 1972; Nicotra et al. 2011), but there are indications that colony form, for example, encrusting versus erect versus free‐living colonies, may also influence zooid size in bryozoans (see Fig. 4.5 in McKinney and Jackson 1989). We hence ask if latitudes and colony form have any explanatory power in accounting for the observed variation among our 1169 species of cheilostomes, regardless of the geological ages of these species. In addition, we investigate the Cope‐Bergmann hypothesis (Hunt and Roy 2006) where an apparent increase in size in younger species could be due to latitudinal shifts, driven by an over‐representation of an out‐of‐the‐tropics (Jablonski et al. 2006) phylogeographical process that has been demonstrated in marine taxa, most notably bivalves (Krug et al. 2009).

## Methods

### DATA

**Figure 1:**
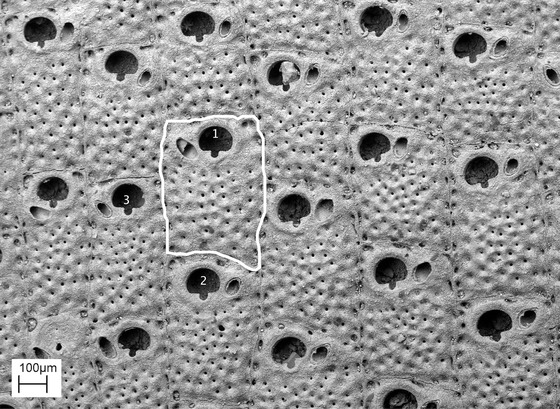
**Scanning electron micrograph of a group of zooids**. A species of *Stylopoma* from the basal Moin Formation (Upper Pliocene 1.9‐1.5 Ma) of Costa Rica from our database that was measured in this work. The three (haphazardly selected) measured zooids are indicated using numbers in the orifices. Outlined in white is the perimeter of zooid number 1 from which the area of this zooid was estimated.

Our raw data consist of scanning electron micrographs (SEMs) of fossil and modern cheilostome bryozoans that PDT has accumulated over several decades of research. They span the entire known geological duration of cheilostome bryozoans (Late Jurassic–Recent) and include specimens from a range of (paleo) latitudes around the globe. Because these digital images were taken for diverse projects unrelated to zooid size documentation, we believe that they are random with respect to zooid size. Criteria for the inclusion of each individual image is that (1) it represents a unique species (in the whole dataset), and (2) the image in question shows at least three measurable autozooids in the zone of astogenetic repetition (i.e., feeding zooids outside the early stages of colony growth where zooids are usually smaller). In each image, three haphazardly selected autozooids were measured using ImageJ (https://imagej.nih.gov/ij/). Only autozooids that are reasonably flat and where the entire outline of the zooid is preserved were selected for measurement (Fig. 1). Area is simply that within the traced outline (Fig. 1). For a haphazard selection of colonies (289 out of 1169, i.e., about 25% of the dataset) in the entire pool of SEMs, we made repeat measurements on the same zooids three times on different occasions (repeating also measurements of the scalebar on the SEM) to estimate the contribution of variation from human measurement errors (Supporting Information Fig. S1).

In addition to area, which is our key size measure, we also measured length (proximal‐distal axis of zooid) and width (greatest width perpendicular to the proximal‐distal axis of zooid) from the same zooids, because these are the most commonly reported zooid size measurements in the bryozoan literature. We were, hence, interested in how well they reflect area, given earlier indications that zooid length and width are not equivalent measurements of size in terms of response to environment factors (O'Dea and Okamura 1999). We show how these three estimates for zooid size are related (Supporting Information Fig. S2) but we focus on presenting results based on area, the parameter that may be most relevant for understanding metabolism (Atkinson et al. 2006), as well as life history variation (Marshall and Monro 2013) and spatial competition (Liow et al. 2017; Liow et al. 2019).

Our specimens are accompanied by taxonomic information, sample details including geographical location and geological age, and colony form. Colonies were identified to species level whenever possible but species that were not formally named were numbered so as to distinguish between congeners. Verbal descriptions of sampling locations were used to approximate present‐day latitudes and longitudes, which were in turn converted to paleolatitudes using the online Paleolatitude Calculator (van Hinsbergen et al. 2015), applying the default settings. The age of each colony was narrowed to the nearest million year using collection specific information (see later sections for how we treated uncertainty in age assignment). Species were categorized into one of four major colony forms: encrusting sheet, encrusting runner, erect, and free living (McKinney and Jackson 1989; Cook et al. 2018b). Our data are available at https://doi.org/10.5061/dryad.nv89424.

### MODELING ZOOID SIZE EVOLUTION

To model temporal patterns, we fitted five standard models of phenotypic change—general random walk (GRW), unbiased random walk (URW), Ornstein‐Uhlenbeck model (OU), Stasis and Strict Stasis (SS) (Hunt 2006; Hunt et al. 2008; Hunt et al. 2015) using the R Package paleoTS (Hunt 2015) to the means of species means in time intervals. Very briefly, the GRW model estimates the mean step and step variance from discrete time intervals, starting from an ancestral state, which is also estimated. This model can detect directional evolution of zooid size. The URW is simply a special case of the GRW where the mean step is zero. This model would fit data where there is no directionality in the evolution of zooid size. The OU model estimates how strongly size is “attracted” (the α parameter) toward a size optimum (θ) given the independent, normally distributed error term. An OU model will best fit data where zooid size did not start at an optimal size but evolved toward an optimum (which is estimated within the model framework, together with an ancestral state). Stasis is modeled as uncorrelated normally distributed variation around a constant and where mean and variance are estimated (Sheets and Mitchell 2001). This may seem similar to the URW model but it does not explicitly consider time steps. Lastly, the SS model is a special case of Stasis where variance is forced to be zero, a scenario we feel is highly unlikely to apply to zooid size, but which we nevertheless fit for completeness.

**Figure 2:**
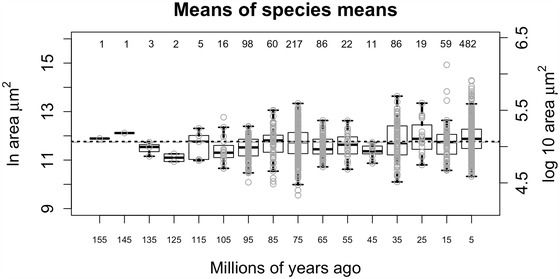
**Autozooid area over time**. Boxplots of means areas of cheilostome species sampled within 10‐million‐year time bins (midpoints are plotted on the *x*‐axis). Both natural and log based 10 are plotted for areas measured in µm^2^. Boxes show interquartile ranges (IQR) while whiskers extend to 1.5 times IQR. Grey open circles are individual species means. Numbers above the boxes are the number of species represented in the given time bin. The dashed gray line is the grand median (11.74) while the dotted black is the grand mean (11.77).

Age estimates of fossils are inherently uncertain, which we handle in three different ways. In the first approach, “Age uncertainty treatment 1,” we binned the nearest million year age estimates in evenly spaced 10 million years time bins, then calculated average sizes from the mean zooid sizes of the species within these time bins (Fig. 2). Note that because variance could not be estimated from the data for the first two 10 million years bins, due to insufficient replicates, we use the mean variance of the rest of the 14 time bins when fitting models of phenotypic change. In the second approach, “Age uncertainty treatment 2,” we simply use the midpoint of the assigned international geological stage (Gradstein et al. 2012) as an estimate for the ages of the fossils, such that, for example, all the colonies found in Tortonian (11.62 to 7.25 Ma) sediments contributed to the pool of Tortonian size data and the mean of their means formed the Tortonian estimate (Supporting Information Fig. S4A). In the third approach, “Age uncertainty treatment 3,” we drew a point estimate from the age range of the geological stage in which a colony was found, by assuming that the probability of the true age of the colony is uniformly distributed within the named stage. We then assigned these drawn ages to equal‐sized 10 myr bins as in “Age uncertainty treatment 1.” After doing so for all the colonies in the dataset, we fitted the five models of phenotypic change and tabulated model support using AICc for each of the 1000 iterations we ran. We report the computed average model support for these 1000 iterations.

### PATTERNS OF ANCESTOR‐DESCENDENT (AD) ZOOID SIZE EVOLUTION

Since we do not have phylogenetic hypotheses based on either morphology or molecules, we used taxonomy and age information for inferring plausible ancestor‐descendent (AD) relationships, a practice that is common when phylogenies are not available for inference (Jablonski et al. 2013). Specifically, we believe that genus identity is a good proxy for close phylogenetic relationships among cheilostome species: recent highly statistically supported molecular phylogenies of cheilostome bryozoans have shown that while higher‐level taxonomies may not always be robust, congeneric species do belong in the same clade, indicating their close evolutionary relationships (Orr et al. 2018). We used genus identity and age information to create AD pairs in two ways.

**Figure 3:**
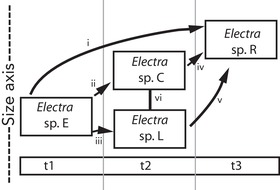
**Assigning and sampling ancestral‐descendent relationships**. Boxes represent colonies of different species belong to the genus *Electra* measured from time intervals t1 (oldest) through t3 (youngest). The vertical direction represents a size axis (not to scale). Roman numbers represent pairs of species while arrows represent assumed relationships (e.g., in “i,” sp. E is older and, therefore, possibly ancestral to sp. C but in “vi” there is not enough information to assign AD relationships for sp. C and L). In “AD pair uncertainty treatment 1,” all relationships i‐v are assumed. See text for AD pair uncertainty treatment 2.

In the first approach, “AD pair uncertainty treatment 1,” we assumed that any older species could have given rise to any (and multiple) younger species in the same genus (Fig. 3 pairs “i” through “v”). For each such pair of species within genera, we tabulated the difference in log mean area and calculated the binomial probability of the descendent species having larger zooids, using all of the differences tabulated across all of the genera (*N* = 128) in which we could carry out this exercise. This gave a total of 3434 putative AD pairs. To generate a null distribution of binomial probabilities here, we randomized areas of colonies within each genus such that these are disassociated from their ages. We then calculated the equivalent binomial probability using this randomized data. Randomization was done 1000 times.

In the second approach, “AD pair uncertainty treatment 2,” which is more conservative, we assumed that a species could only give rise to one descendent in the same genus, which we randomly assigned from a younger species assigned to that genus. Note that this is inspired by the treatment used by Alroy (1998). When there were multiple plausible descendants within the genus, one or more descendants may be left out in any given iteration of such a random selection. Therefore, we implemented this repeatedly, selecting AD pairs within genera without replacement in each iteration (e.g., “i” in Fig. 3 is the only pair that is selected in one iteration; “ii” and “iv” in another; and “iii” and “v” in another). The bootstrapped binomial probability of 1000 iterations is presented and compared with a null model in which age is disregarded in randomly paired species within a genus (also repeated 1000 times).

Note that we removed all species of unknown and uncertain generic identity (i.e., those genera labeled “cf.” and “?” in the original database) from these AD analyses to eliminate ambiguity.

### THE CONTRIBUTION OF LATITUDE, COLONY FORM, MEASUREMENT ERROR, AND SPECIES RANDOM EFFECTS TO ZOOID SIZE

As mentioned in the Introduction, latitude is known to influence zooid size among species, whereby species that occur at high latitudes often have larger zooid sizes than their congeners in lower latitudes (Kuklinski and Taylor 2008). To test if latitude can explain variation in zooid size across species, we first ask how much measurement error and interspecies differences explain the variation that we have documented in our samples. We modeled log area for the subset of zooids in which repeat measurements were taken, using both species identities and repeat measurements as random effects. Because we found that measurement error contributed little to the variation in zooid areas (see Results and Table S2) and that the model including only species random effects was statistically indistinguishable from one with both repeated measurements and species random effects, we treated the rest of the dataset by including species identity as a random effect in the model. We constructed models of log zooid size using combinations of latitude and colony form as explanatory variables (fixed effects) and compared these models using AIC and also estimated the contributions of these variables. We implemented these linear mixed effects models using the R Package lme4 (Bates et al. 2015).

### THE CONTRIBUTION OF LATITUDINAL SHIFTS TO MODULE SIZE CHANGES FROM ANCESTOR TO DESCENDENT

To investigate the contribution of Bergmann's Rule to Cope's Rule (as was found to be the case for deep‐sea ostracodes, see Hunt and Roy 2006) we studied the relationship between log size differences and latitudinal shifts (between putative AD pairs in “AD pair uncertainty treatment 1”). More specifically, we modeled log size differences as a function of latitudinal changes and/or the amount of time that has passed between the ancestor and the descendent (as fixed effects) while using genus identity as random effects. As before, we implemented these linear mixed effects models using the R Package lme4 (Bates et al. 2015). Our code is available at https://doi.org/10.5061/dryad.nv89424 and all analyses are run in R version 3.5.2 (R Core Team 2018).

## Results

The grand median autozooid size for our entire dataset is 1.25 × 10^5^ ± 1.73 × 10^5^ µm^2^ or 11.74 in natural log, the unit we use for presenting the rest of our results (see Supporting Information Fig. S3). There is substantial variation in how much autozooid size varies within a given species but measurement error contributes little to this variation (see Supporting Information Fig. S1 and Table S2). There is also substantial variation among species that spans an order of magnitude (Supporting Information Fig. S1). The very first cheilostomes were above average in zooid areas and the species means undulate across 150 million years of their history (Fig. 2 and Supporting Information Fig. S4).

Fitting the five evolutionary models to species averages in 10 million years bins (i.e., data from Fig. 2 using “Age uncertainty treatment 1”), we found that Stasis had the highest model weight (57%) followed by an OU model (31%, see Supporting Information Table S1 for weights of other models). The parameter estimates for the stasis model are *θ* = 11.67 and *ω* = 0.02, while those from the OU model are ancestral size = 11. 31, step variance = 1^−10^, *θ* = 14.6, *α* = 0.001. Stasis remains the best model (81%) when geological stages are used, that is, “Age uncertainty treatment 2” (see Supporting Information Table S1). In “age uncertainty treatment 3,″ both a SS model and GRW perform poorly, as in the first two age uncertainty treatments, while model weights are more evenly distributed between the URW, OU, and Stasis models (Supporting Information Table S1).

**Figure 4:**
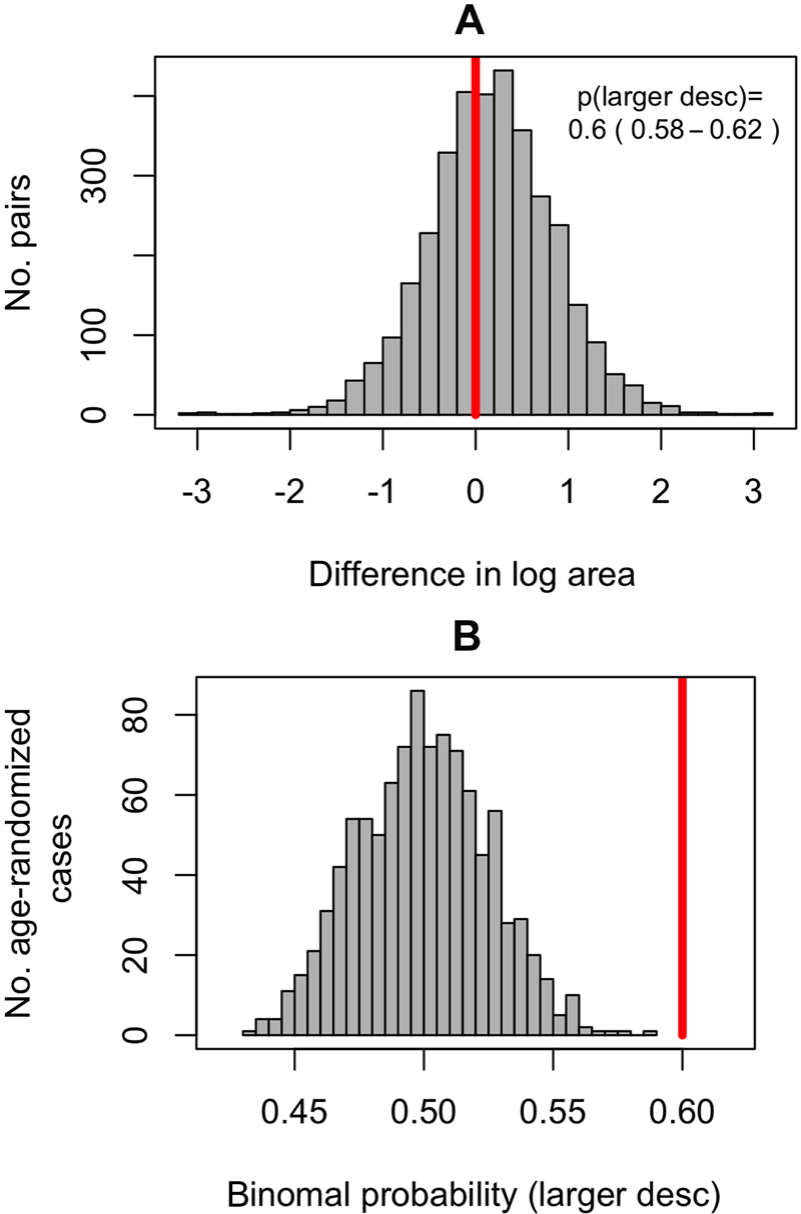
**Size differences between putative ancestor‐descendent (AD) pairs**. (A) Shows a histogram of the differences between mean ln area (µm^2^) of all putative AD pairs. The red line is placed at 0 (no difference). The binomial probability of putative descendants being larger is 0.6 (CI: 0.58‐0.62, *N* = 3434). (B) Shows the distribution of the same binomial probability (1000 iterations) when the ages of species within genera are randomized. In (B), the red line is plotted at 0.6 (the estimate from the original data).

**Figure 5:**
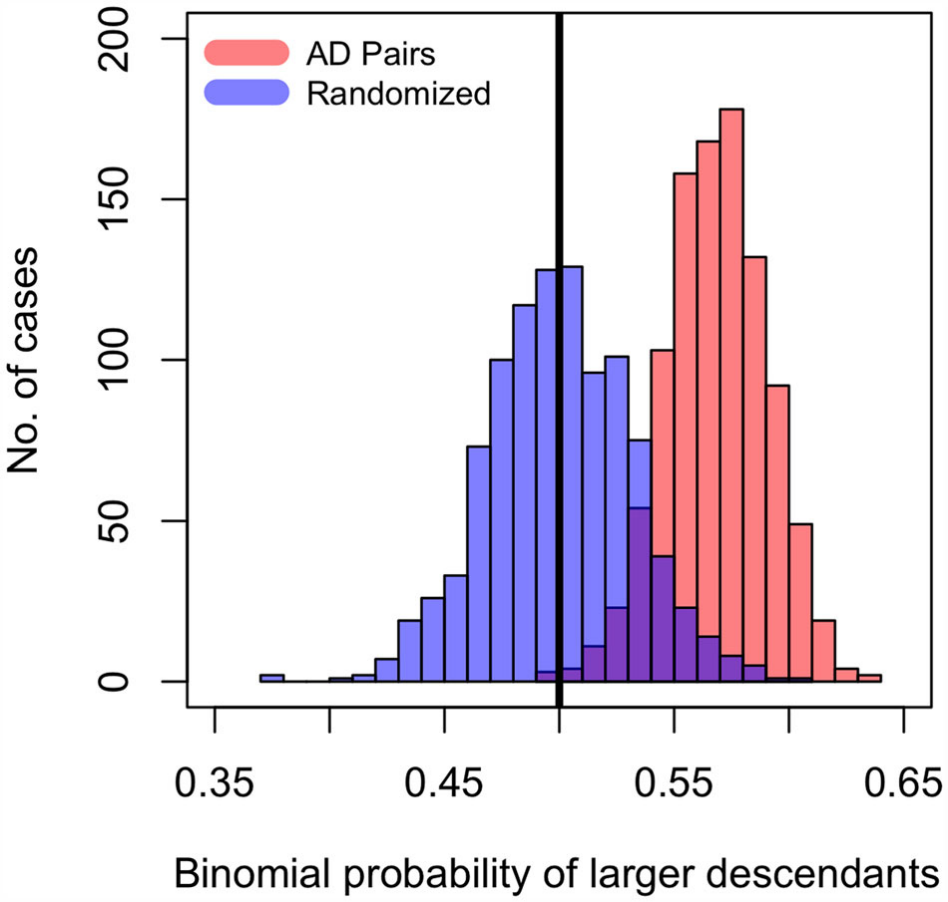
**Probabilities of descendants having larger autozooids**. The red histogram are binomial probabilities of younger species being larger in ln area in randomized AD (i.e., older and younger) pairs sampled without replacement within genus. The blue histogram is the null distribution of binomial probabilities based on the empirical dataset, where species pairs are drawn with no regard to their age. The black vertical line is draw at the mean of the theoretical null expectation.

By assuming any older species can be the ancestor of any younger species in the same genus (“AD pair uncertainty treatment 1”), we found that the binomial probability of a descendent having larger zooids is 0.6 (95% confidence interval 0.58‐0.62, Fig. 4). When we assume that a species can only give rise to one (randomly selected) descendent in the same genus (i.e., “AD pair uncertainty treatment 2”), the binomial probability of a larger descendent from 1000 iterations of randomly assigned descendants is decidedly greater than 0.5 and also very different from a null distribution generated from our data that disregards temporal order (Fig. 5, two‐sample Kolmogorov‐Smirnov test, *P* < 2.2 × 10^−16^). Looking at the averages of binomial probabilities in each genus, we also see that in more than half of the genera, the average probability of having descendants larger than their ancestors is greater than the null of 0.5 (Supporting Information Fig. S5), this being more pronounced in more data‐rich genera such as *Microporella* and *Onychocella*.

Using all of the species for which the randomly selected autozooids have been measured three times each, we found that measurement error accounted for substantially less variance (0.016) than interspecies variation (0.72, see Supporting Information Table S2 and Fig. S1). While a model that uses both random effects from measurement error and species identity performs better (by an AIC criterion, Supporting Information Table S2) for the subset of our data for which we have repeated measurements, we note that the second‐best model (with only species random effects) has very similar fixed effect and random effect estimates. For completeness, we studied two different models that include time intervals: neither the model with time as a random effect or as a fixed effect improves the basic species random effects model (Supporting Information Table S2). The model with time as a random effect performed better than that with time as a fixed effect.

Since we did not undertake repeat measurements for all the colonies, we used a species random effects model as a base model for exploring the relationship between zooid size, latitude, and colony form, after checking that the estimates (coefficients) for the base model were similar for the data subset as the full dataset. We found that the best model is the one including only (paleo)latitude as a fixed effect, when only two most common colony forms are included in the analyses. Here, increasing latitude by one degree increases zooid size by 0.012 natural log units (Supporting Information Table S3). When all four colony forms are included in the analyses, the model including colony forms and latitude is deemed the best, but differences between colony forms are estimated with large error due to small samples sizes (Supporting Information Table S3). Here, zooid sizes in encrusting sheets are larger than those in erect colonies, whereas those in encrusting runners and free‐living colonies are somewhat smaller than those in erect colonies, and the effect of latitude is as is predicted by the simpler model using only the two most common colony forms (encrusting sheets and erect colony forms).

To test the possibility that the AD patterns might be an artifact of older species (putative ancestors) from lower latitudes giving rise to younger species (putative descendants) that moved into higher latitudes (i.e., an “out of the tropics” model of speciation) and, hence, had larger zooids, we also modeled changes in zooid size in AD pairs as a function of the change in latitude using all the AD pairs we could generate (“AD pair uncertainty treatment 1”). The model with log size changes modeled as genus random effects performed best, although all three models (Supporting Information Table S4) are minimally different from each other; hence, we can draw the conclusion that change in latitude did not contribute to the AD pattern (see also Supporting Information Fig. S6).

## Discussion

Cope's Rule of phyletic size increase, if observed at the level of the entire clade as in brachiopods (Novack‐Gottshall and Lanier 2008) and canid mammals (van Valkenburgh et al. 2004), for instance, can be driven by at least three distinct and nonmutually exclusive processes. First, it could happen simply as a result of clades arising from small‐sized ancestors (Stanley 1973), via what is now called passive diffusion (McShea 1994). Second, species can also increase in size over time due to selection at the level of the individual (Kingsolver and Pfenning 2004). Last, species that have small sizes can have higher extinction rates and/or those with larger sizes can have higher speciation rates (Jablonski 2017).

Passive diffusion from the “left wall” of small size can in principle explain the increases in zooid size, although Stanley's idea of smaller organisms being less specialized (Stanley 1973) has not yet been systematically evaluated in cheilostomes. However, we note that the very earliest cheilostomes (the two Jurassic species belonging to the genus *Pyriporopsis*) have zooids that are of larger rather than smaller than the grand average for cheilostomes over 150 million years (Fig. 2 and Supporting Information Fig. S4), meaning that Stanley's macroevolutionary mechanism does not furnish a viable explanation for the pattern of zooid size changes in this group as a whole. It remains to be seen if this can be true at the level of subclades, but we have not sampled any genera comprehensively enough to be able to test if the first species in any given genus has smaller zooids in general. As multiple SEMs of colonies of different geological ages for a given species were not available for this study, we could not evaluate the second proposed process. Cope's Rule holds for cheilostome bryozoans at the level of the module (zooid), at least when putative ancestor‐descendants are analyzed (the third process above) but we did not evaluate the third process again due to the absence of suitable data. All things considered, our results do not contradict the finding that there is little detectable selective advantage of size increase in contemporary populations (Gotanda et al. 2015). However, they do suggest that any microevolutionary processes cannot easily be extrapolated to macroevolutionary patterns without knowing more about detailed selective and/or “passive” mechanisms of evolution at different time scales.

Given that we have detected a tendency for module size increase within subclades, using data from more than a thousand cheilostome species, it is worth contemplating what the biological mechanisms driving this could be. How might explanations proposed for unitary organisms be applied to the increase in zooid size shown in the present study? With regard to individual selective advantage, large size in unitary organisms (see Newall 1949 and Stanley 1973 for more comprehensive reviews) has been suggested as advantageous in: (1) escape from predators (Benton 2002), (2) prey capture (Benton 2002), (3) trophic competition (Bonner 1988); (4) competition and resource use for other resources (Brown and Maurer 1986); (5) resistance to environmental stress (Peters 1983), (6) social dominance including competition for mates (Andersson 1994), (7) fecundity (Andersson 1994), (8) extended longevity (Brown and Sibly 2006), and (9) increased heat retention (Stanley 1973). Explanations (6) and (9) can be eliminated immediately in the sessile ectothermic bryozoans. It is difficult to envisage how large zooid size could help in avoiding predation (1), even for the micropredators that consume single zooids at a time (Lidgard 2008). We know of no data directly correlating zooid size with either fecundity (7) or longevity (8). Resistance to environmental stress (5) could potentially correlate with zooid size given the lower surface area/volume ratio of larger zooids but, again, empirical data to support this idea are not currently available. With regard to feeding (2), larger zooids presumably create stronger currents for suspension feeding and may have mouths of larger diameter (McKinney 1993), raising the possibility that they can capture and consume larger and more motile prey items. Stronger currents may also be advantageous in competition for food (3) when this is a limiting resource (see e.g., Buss 1979). While this requires testing, large zooid size has been shown to be a factor in competition for a different resource (4), living space among encrusting cheilostomes (Liow et al. 2017, 2019). Resource competition, both for food and space, is currently the most compelling explanation for zooid size increase through time. Our data also demonstrated that zooid size is very variable among species. This variation cannot be explained by measurement error, colony form, or geological age for our dataset, but is detectably influenced by the latitude at which the bryozoan lives or lived. Seasonal variations within single bryozoan colonies have been observed, where zooids budded in warmer waters during the summer have smaller zooids that those budded in colder conditions during the winter (O'Dea and Jackson 2002). In fact, bryozoan colonies have even been used to infer paleoseasonality using this relationship (Knowles et al. 2009). It has also been found that species with larger zooids are more often found living in higher latitudes (Kuklinski and Taylor 2008) or at lower temperatures (Okamura and Bishop 1988). It is tempting to speculate that paleotemperatures, rather than geological age, may have contributed to our pattern for zooid size (see Hunt and Roy 2006 for a parallel phenomenon in deep‐sea ostracodes). There has been a general cooling trend in the Cenozoic (Zachos et al. 2001), and given the association between temperature and zooid size, this global trend may have contributed to part of the pattern we see (Fig. 2) but because we only have 16 time points, we cannot justify studying causal relationships between macroevolutionary patterns of size and global temperature more closely until we have more and/or different types of data (Hannisdal and Liow 2018).

The pattern of zooid sizes through the late Mesozoic to the Recent does not conform easily to standard models of phenotypic change, even though the models with best fit implied a relatively stable zooid size. Our data show that many earlier Cretaceous bryozoans were small (Fig. 2) but that many later Cretaceous ones were larger. Then, after the Cretaceous‐Paleogene boundary (KPg, 66 Ma), zooid sizes again dipped below the grand average (Fig. 2 and Supporting Information Fig. S4), before showing a general increase, albeit with a lot of variation, toward the Recent. While a Lilliput effect (Urbanek 1993), described as a decrease in body size across a mass extinction event, is often found in marine invertebrate groups and is thought to be driven by environmental changes accompanying these events, it appeared to be absent in a handful of bryozoans that crossed the KPg extinction boundary (Sogot et al. 2014). Our current results, based on a richer dataset, suggest otherwise, although we did not explicitly track lineages that cross this mass extinction boundary.

As in all empirical studies, we have to recognize the limitations of our dataset and approaches. First, there are about 5000 described species of living cheilostomes (Bock and Gordon 2013), and probably many more undescribed ones, both extinct and extant, and we have only sampled a small proportion of these. However, as mentioned earlier, we see no reason why these species represent a biased sample with regards to our trait of interest, which is zooid size. Second, within‐colony zooid size variation is present in any given bryozoan (e.g., Supporting Information Fig. S1), and so is among colony variation for a given species (Liow et al. 2017), and we have represented this variation with only one colony per species. But we note that the within‐colony variation we observed in our dataset is well within the limits of the variation within a given species that we see across a substantial amount of time (Supporting Information Fig. S1). Third, measurement error can contribute to variation in our observations and is not something that is commonly tackled in the literature for Cope's Rule or the bryozoan literature. But, we have clearly shown that measurement error is not a concern in our dataset (Supporting Information Table S2). Fourth, both latitude and geological age data come with a substantial amount of uncertainty, but we have tackled the uncertainty in age in several different ways, only to draw the same conclusions with respect to our questions. Fifth, more serious than species sampling, errors of measurement and size estimation, and uncertainties in age and latitude, are our lack of direct information for ancestral‐descendant relationships. We approximate this in several different ways, but without an independently constructed and robust phylogeny, we cannot know for certain that these inferred relationships accurately represent genealogy. However, Foote (1996) showed that is a good chance of sampling both direct and indirect ancestors in the fossil record for taxa with relatively good fossil record. The fossil record of cheilostomes is rich and even though our sampling is not exhaustive, we believe that at least some of our AD pairs are “real,” even if they are not all “direct.” Last, we only attempted to infer AD relationships within genus but completely neglected AD relationships contributed by a species of a new genus that originated from a different genus. Including such information in the future will allow us to address the hypothesis that new clades may start small, as suggested by Stanley (1973).

Using an unprecedented dataset of cheilostome zooid sizes, we find a pattern of increases in zooid sizes within subclades although the whole‐clade pattern of zooid sizes through time suggest imprints of global climate change, extinction events, and broad macroecological patterns (Bergmann's Rule). Simultaneously, zooid size seems rather stable, which in turn suggest that there is an optimal zooid size, not just for a given species (Marshall and Monro 2013), but also for cheilostomes in their entirety. The different levels of agreement of empirical data with Cope's Rule among unitary organisms cautions us against extrapolating theories developed for unitary to colonial organisms. Module size is a deceptively simple trait, but many forces act in concert to constrain or change it. Only with detailed analyses using global, regional, and local data on different temporal and spatial scales (Berke et al. 2013) can we begin to understand the ecological and evolutionary mechanisms underlying the patterns we see. Different selective and passive forces are revealed in microevolutionary and macroevolutionary approaches and efforts to evaluate these in concert are needed before we can begin to understand the links between them.

Associate Editor: P. Wagner

Handling Editor: M. R. Servedio

## Supporting information


**Fig. S1**. Zooid area measurements for each species.
**Fig. S2**. Length, width, area relationships.
**Fig. S3**. Distribution of ln zooid area.
**Fig. S4**. Autozooid area over time.
**Fig. S5**. Average probabilities of larger descendants within genera.
**Fig. S6**. Shift in latitude versus change in ln zooid size (from ancestor to descendent).
**Table S1**. Phenotypic change model weights.
**Table S2**. A comparison of basic random effects models.
**Table S3**. A comparison of mixed models including paleolatitude and growth forms.
**Table S4**. A comparison of mixed models for change in area from ancestor to descendent.
