# Supplementary material for: Cope's Rule in a modular organism: Directional evolution without an overarching macroevolutionary trend
Source: Evolution. 2019 Jul 23;73(9):1863–72. doi: 10.1111/evo.13800 (PMC6771556; doi:10.1111/evo.13800)

# Supplementary material for MS (Evolution)

# Cope's Rule in a modular organism: Directional evolution without an overarching macroevolutionary trend

**Fig. S1. Zooid area measurements for each species.** Natural log (ln area μm$^2$) of the three haphazardly selected zooids for each named image on the x-axis are shown. Repeat measurements of the areas of select colonies as shown as red (second measurement) and blue (third measurement) dots where available. Note that there is no temporal order within each of the named geological time interval (as assigned in the original metadata of the image files) and some have multiple plots. On the first plot we also show the median and interquartile range of measurements from 180 zooids each from six time intervals (ranging from 2.3 Ma to 0 Ma) of six species from New Zealand (data from Liow et al. 2017). S. mag = *Steginoporella magnifica;* A. mar = *Aimulosia marsupium;* C. cri. = *Crepidacantha crinispina;* M. ago = *Microporella agonistes;* V. val = *Valdemunitella valdemunita*; C .ang = *Calloporina angustipora.* The second time interval is simply labeled 'pre-Albian' because we only have 8 cheilostomes for several geological stages (from the Berriasian to the Aptian, i.e. 145 to 113 million years ago).

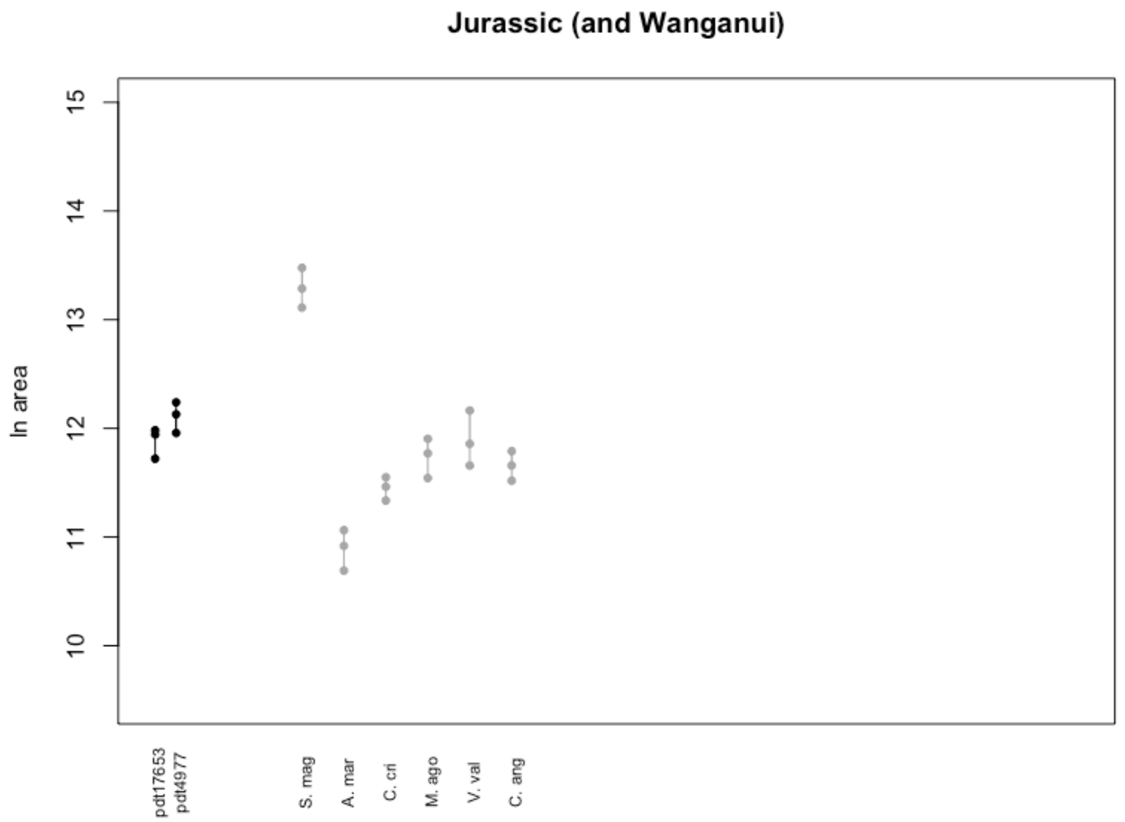

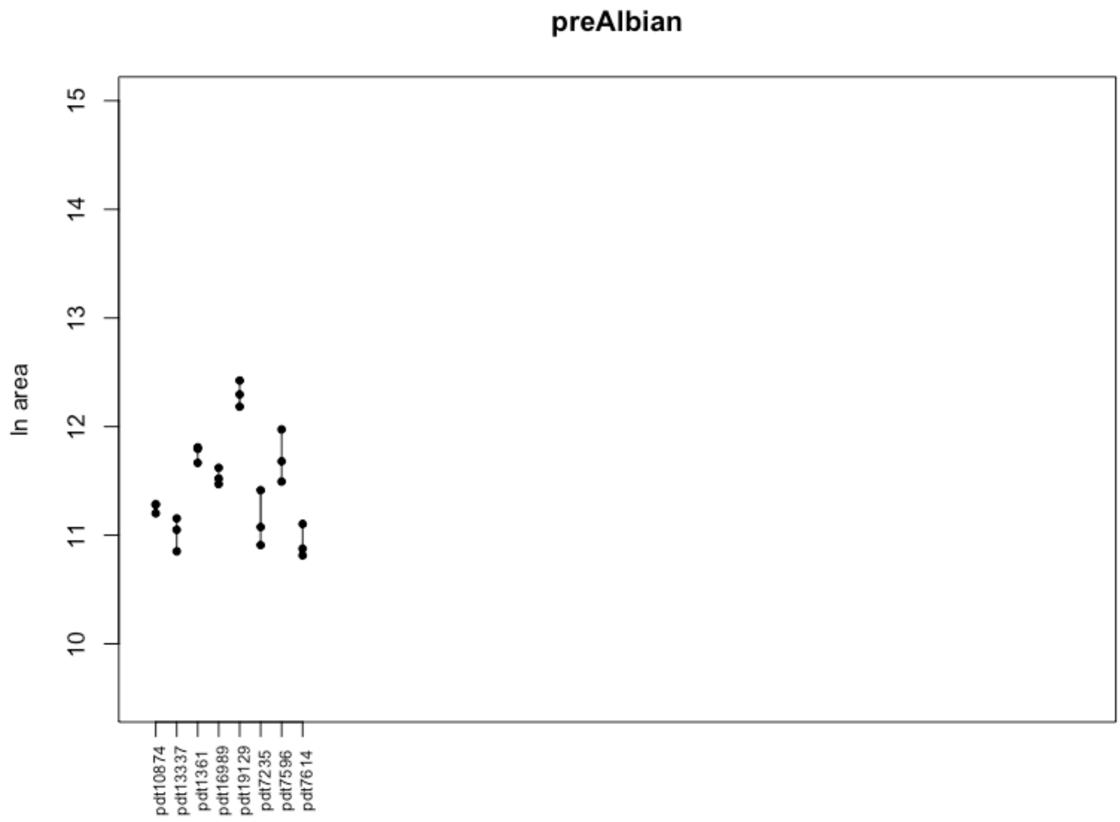
preAlbian
ln area
10
11
12
13
14
15
pdt10874
pdt13337
pdt1361
pdt16989
pdt19129
pdt7235
pdt7596
pdt7614

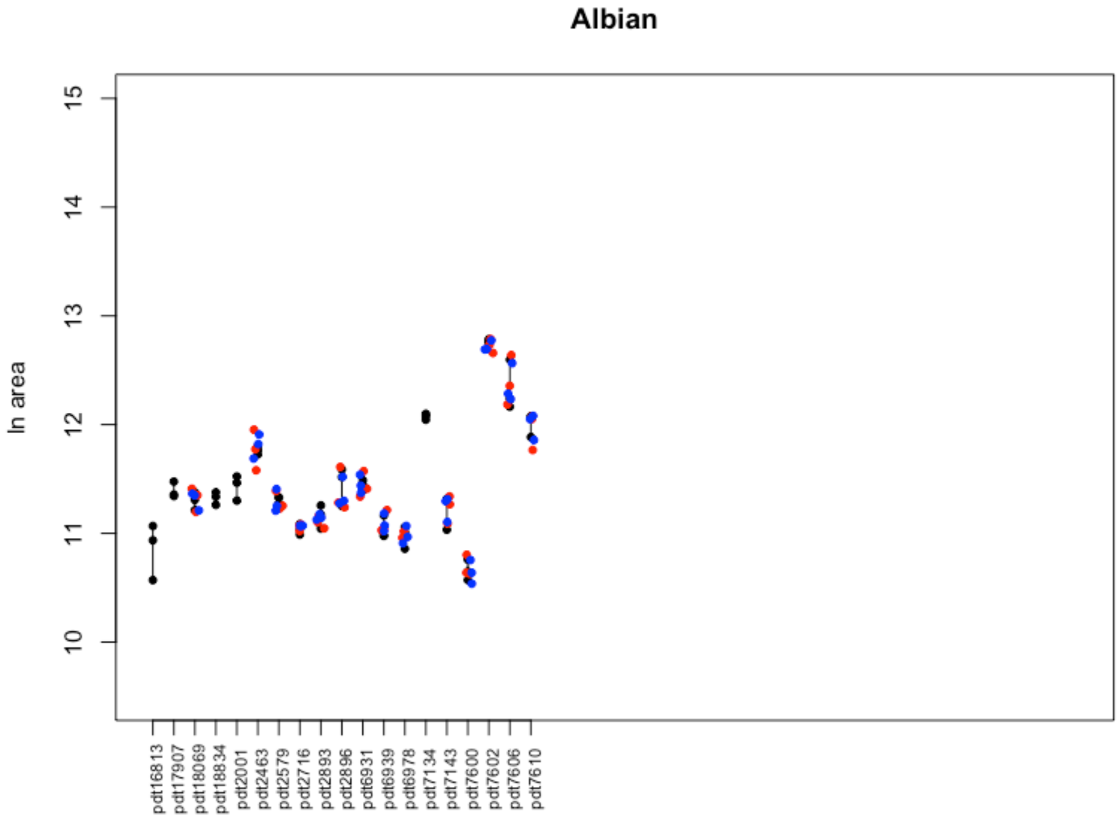
Albian
ln area
10
11
12
13
14
15
pdt16813
pdt17907
pdt18069
pdt18834
pdt2001
pdt2463
pdt2579
pdt2716
pdt2893
pdt2896
pdt6931
pdt6939
pdt6978
pdt7134
pdt7143
pdt7600
pdt7602
pdt7606
pdt7610

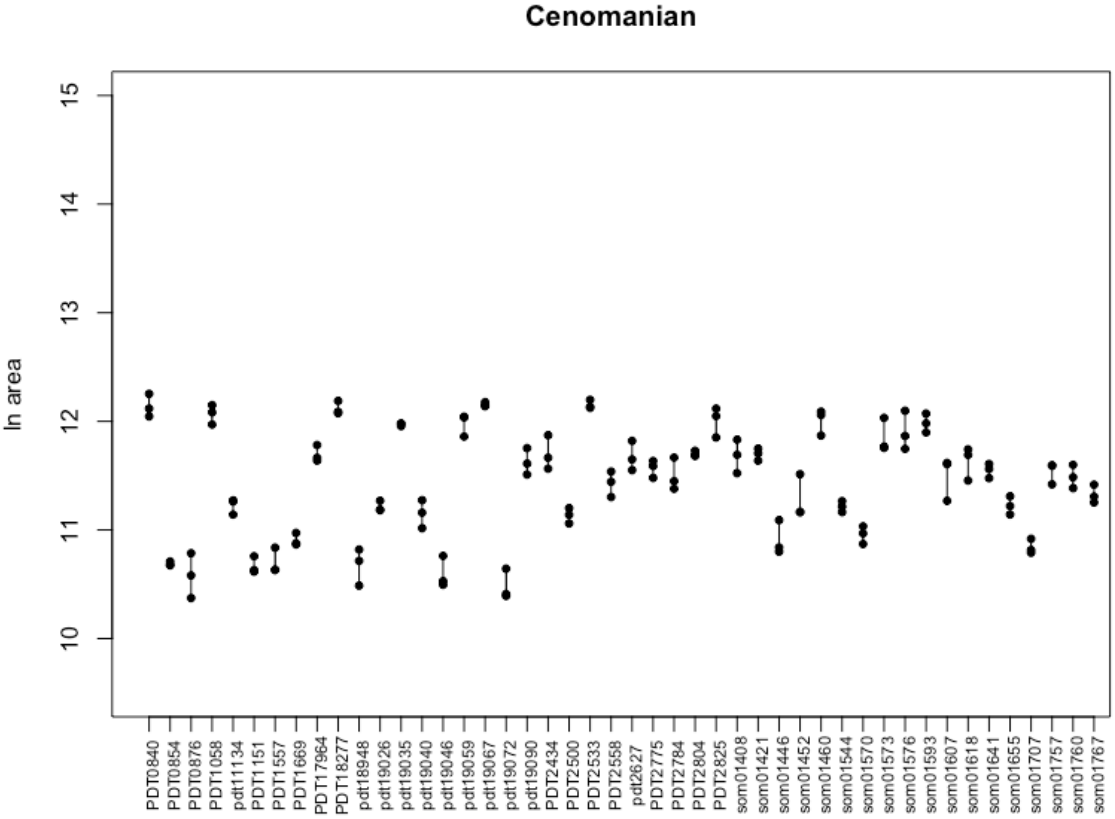
Cenomanian
ln area
15
14
13
12
11
10
PDT0840
PDT0854
PDT0876
PDT1058
pdt11134
PDT1151
PDT1557
PDT1669
PDT17964
PDT18277
pdt18948
pdt19026
pdt19035
pdt19040
pdt19046
pdt19059
pdt19067
pdt19072
pdt19090
PDT2434
PDT2500
PDT2533
PDT2558
pdt2627
PDT2775
PDT2784
PDT2804
PDT2825
som01408
som01421
som01446
som01452
som01460
som01544
som01570
som01573
som01576
som01593
som01607
som01618
som01641
som01655
som01707
som01757
som01760
som01767

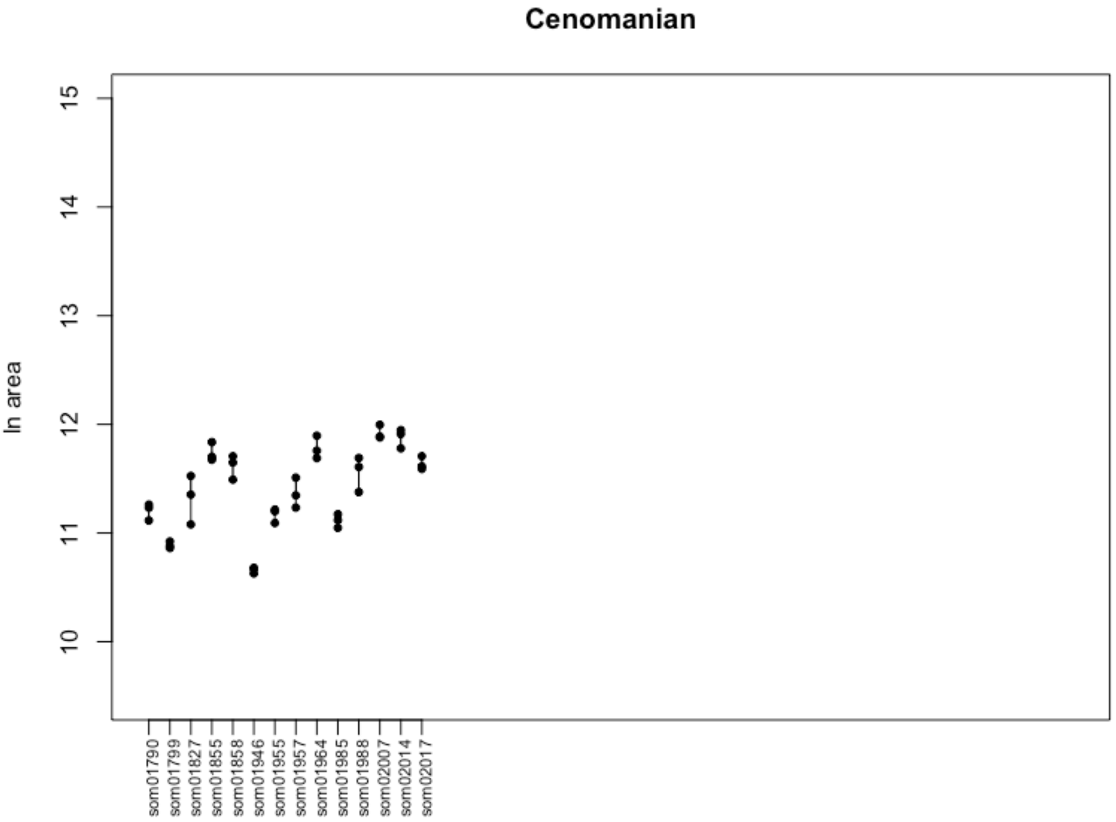
Cenomanian
ln area
15
14
13
12
11
10
som01790
som01799
som01827
som01855
som01858
som01946
som01955
som01957
som01964
som01985
som01988
som02007
som02014
som02017

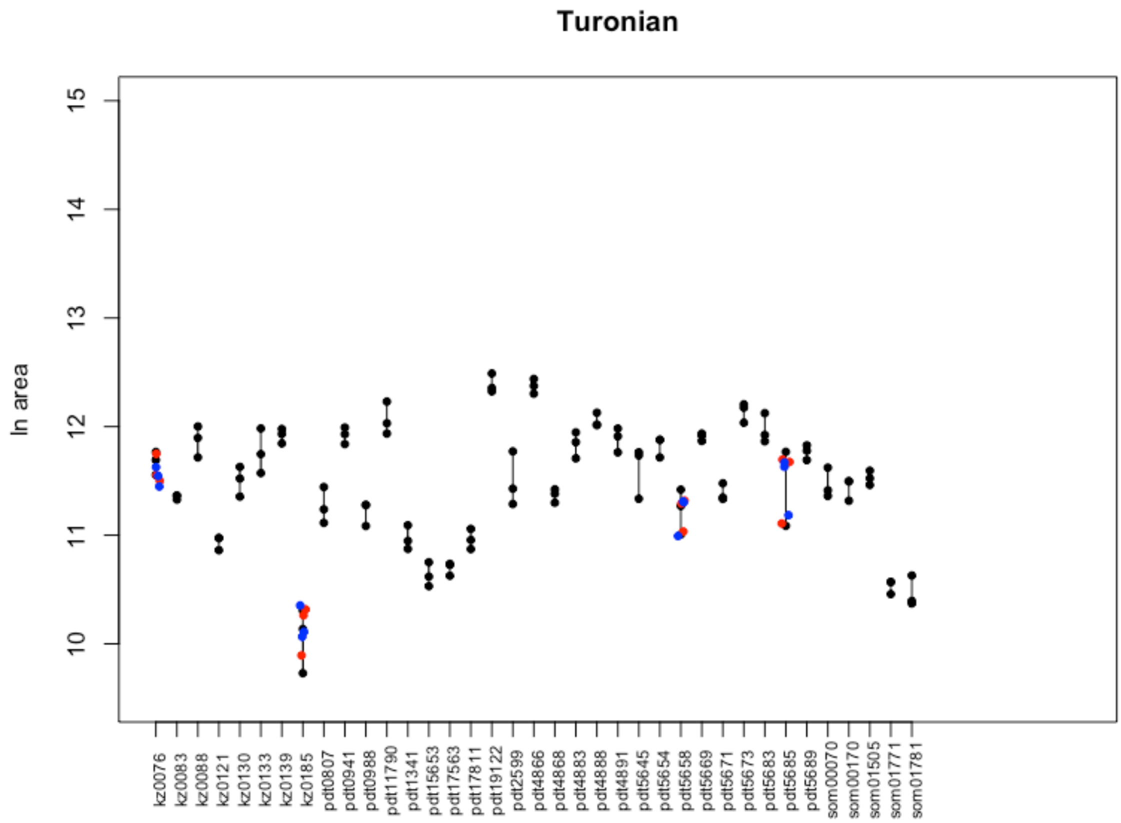
Turonian
ln area
15
14
13
12
11
10
kz0076
kz0083
kz0088
kz0121
kz0130
kz0133
kz0139
kz0185
pdt0807
pdt0941
pdt0988
pdt11790
pdt1341
pdt15653
pdt17563
pdt17811
pdt19122
pdt2599
pdt4866
pdt4868
pdt4883
pdt4888
pdt4891
pdt5645
pdt5654
pdt5658
pdt5669
pdt5671
pdt5673
pdt5683
pdt5685
pdt5689
som00070
som00170
som01505
som01771
som01781

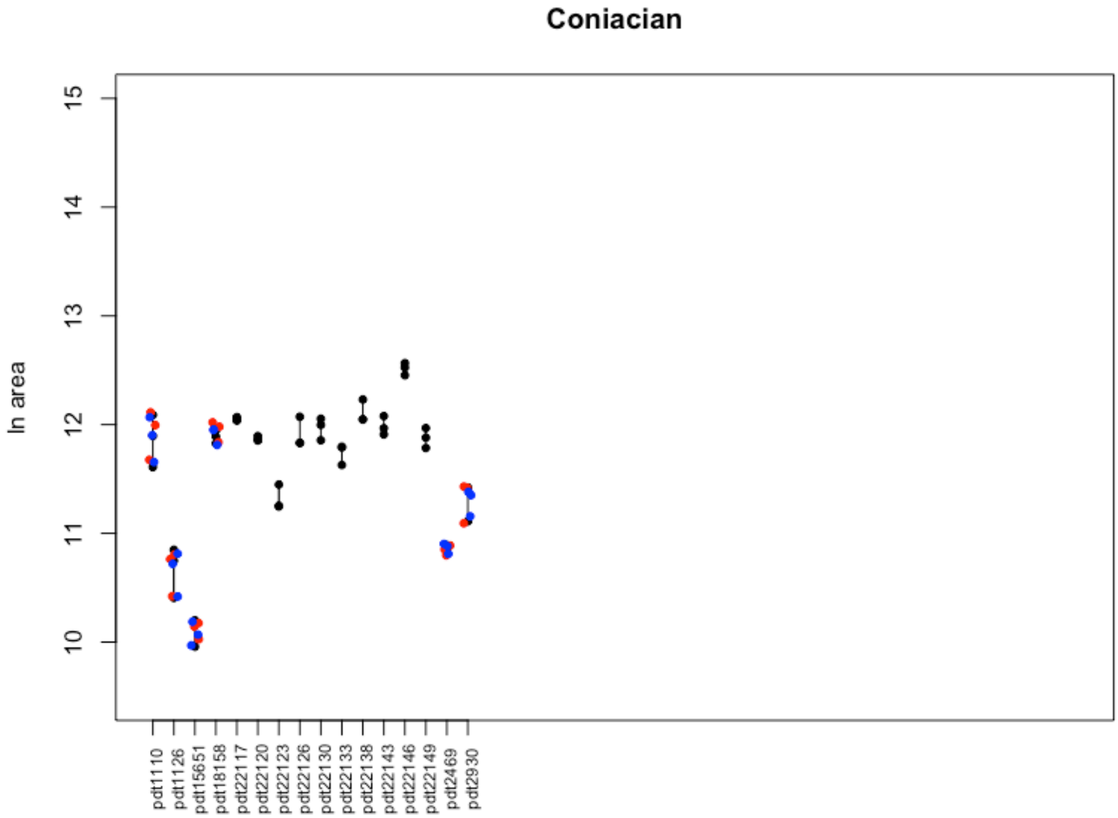
Coniacian
ln area
15
14
13
12
11
10
pdt1110
pdt1126
pdt15651
pdt18158
pdt22117
pdt22120
pdt22123
pdt22126
pdt22130
pdt22133
pdt22138
pdt22143
pdt22146
pdt22149
pdt2469
pdt2930

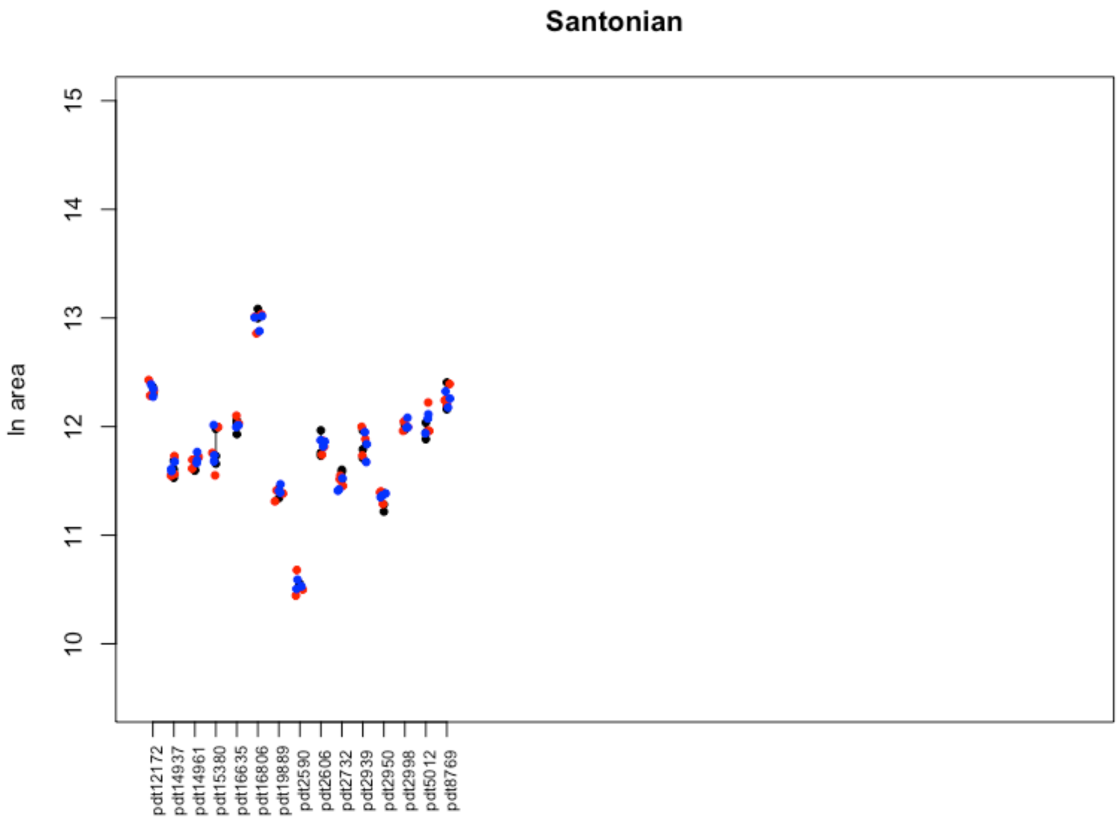
Santonian
ln area
15
14
13
12
11
10
pdt12172
pdt14937
pdt14961
pdt15380
pdt16635
pdt16806
pdt19889
pdt2590
pdt2606
pdt2732
pdt2939
pdt2950
pdt2998
pdt5012
pdt8769

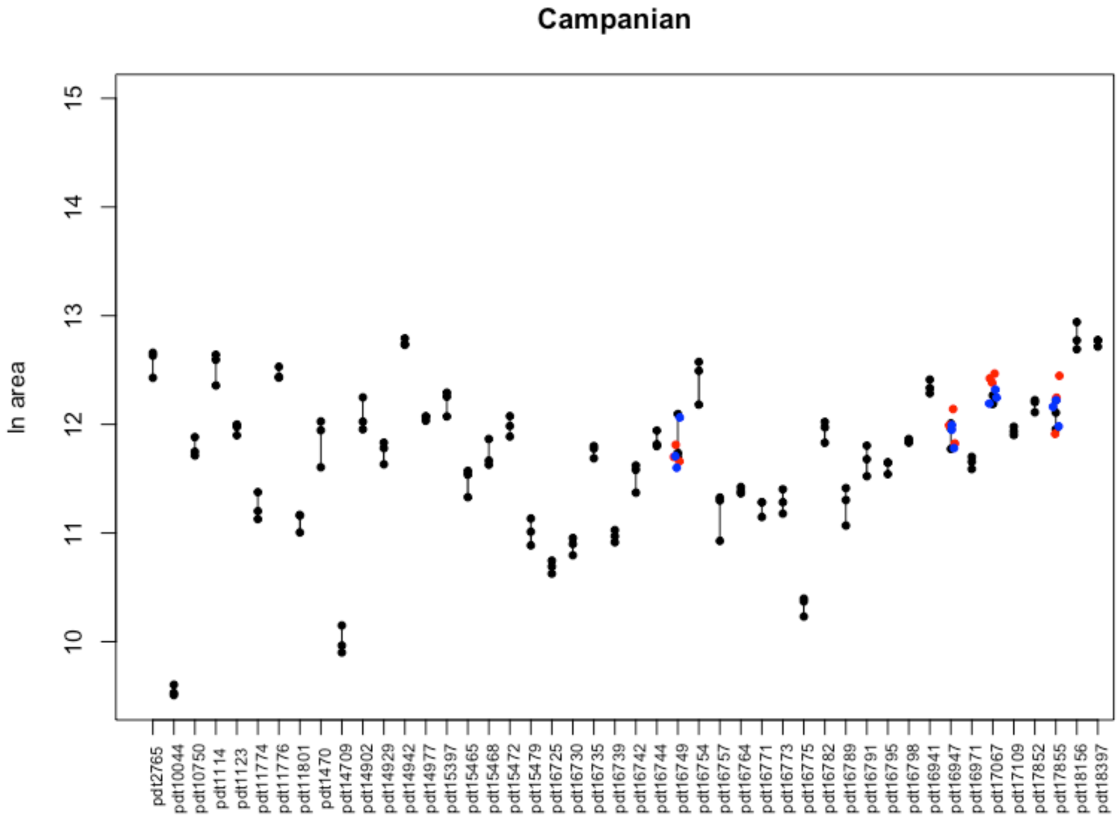
Campanian
ln area
15
14
13
12
11
10
pdt2765
pdt10044
pdt10750
pdt1114
pdt1123
pdt11774
pdt11776
pdt11801
pdt1470
pdt14709
pdt14902
pdt14929
pdt14942
pdt14977
pdt15397
pdt15465
pdt15468
pdt15472
pdt15479
pdt16725
pdt16730
pdt16735
pdt16739
pdt16742
pdt16744
pdt16749
pdt16754
pdt16757
pdt16764
pdt16771
pdt16773
pdt16775
pdt16782
pdt16789
pdt16791
pdt16795
pdt16798
pdt16941
pdt16947
pdt16971
pdt17067
pdt17109
pdt17852
pdt17855
pdt18156
pdt18397

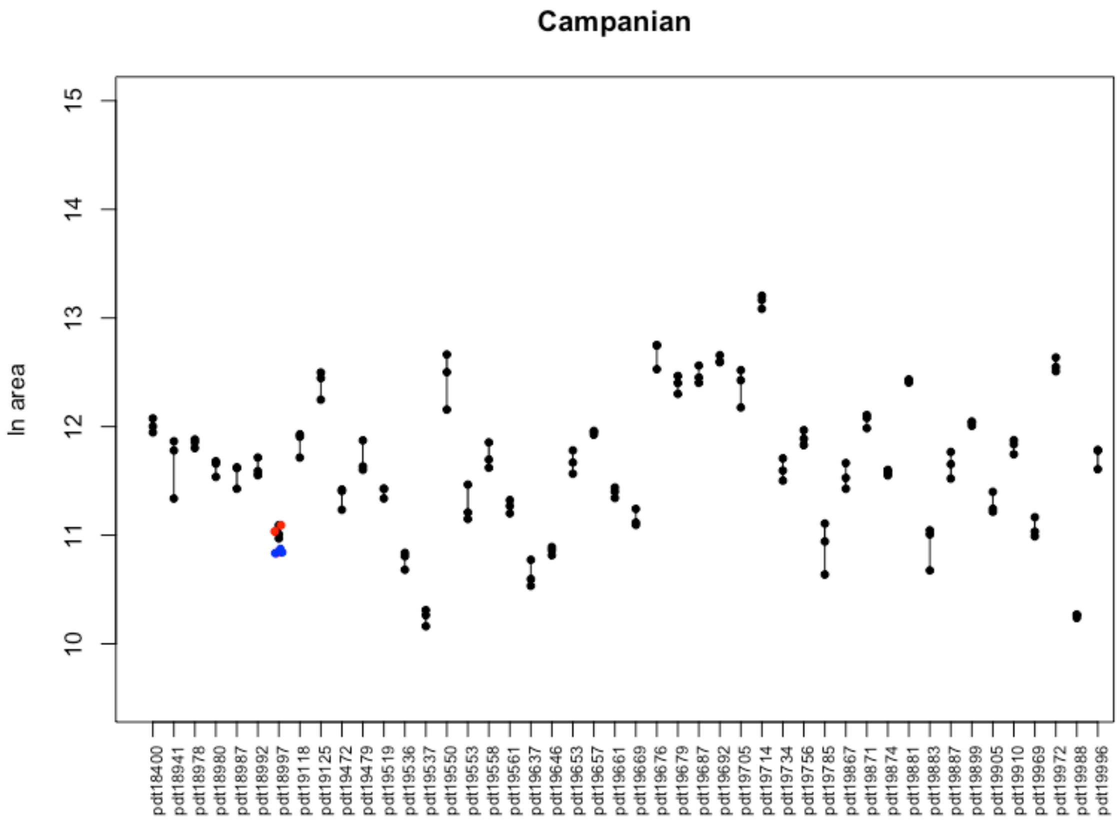
Campanian
ln area
15
14
13
12
11
10
pdt18400
pdt18941
pdt18978
pdt18980
pdt18987
pdt18992
pdt18997
pdt19118
pdt19125
pdt19472
pdt19479
pdt19519
pdt19536
pdt19537
pdt19550
pdt19553
pdt19558
pdt19561
pdt19637
pdt19646
pdt19653
pdt19657
pdt19661
pdt19669
pdt19676
pdt19679
pdt19687
pdt19692
pdt19705
pdt19714
pdt19734
pdt19756
pdt19785
pdt19867
pdt19871
pdt19874
pdt19881
pdt19883
pdt19887
pdt19899
pdt19905
pdt19910
pdt19969
pdt19972
pdt19988
pdt19996

33

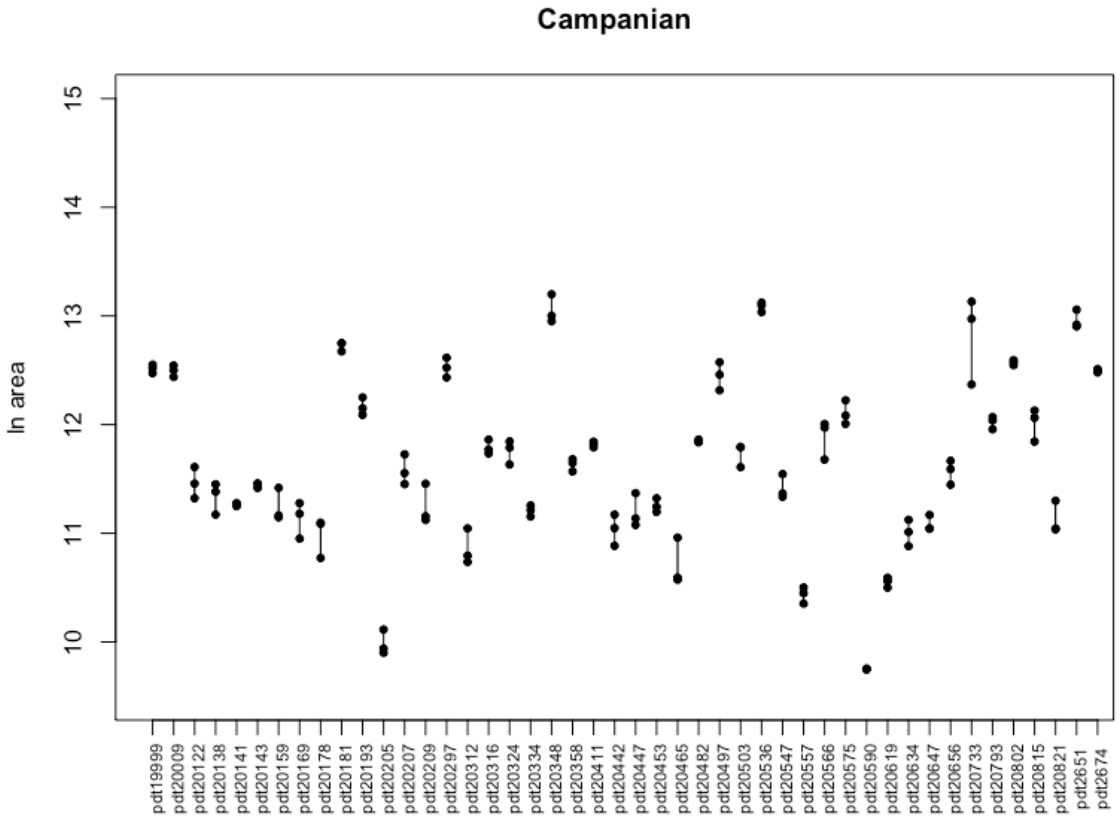
Campanian
ln area
15
14
13
12
11
10
pdt19999
pdt20009
pdt20122
pdt20138
pdt20141
pdt20143
pdt20159
pdt20169
pdt20178
pdt20181
pdt20193
pdt20205
pdt20207
pdt20209
pdt20297
pdt20312
pdt20316
pdt20324
pdt20334
pdt20348
pdt20358
pdt20411
pdt20442
pdt20447
pdt20453
pdt20465
pdt20482
pdt20497
pdt20503
pdt20536
pdt20547
pdt20557
pdt20566
pdt20575
pdt20590
pdt20619
pdt20634
pdt20647
pdt20656
pdt20733
pdt20793
pdt20802
pdt20815
pdt20821
pdt2651
pdt2674

34

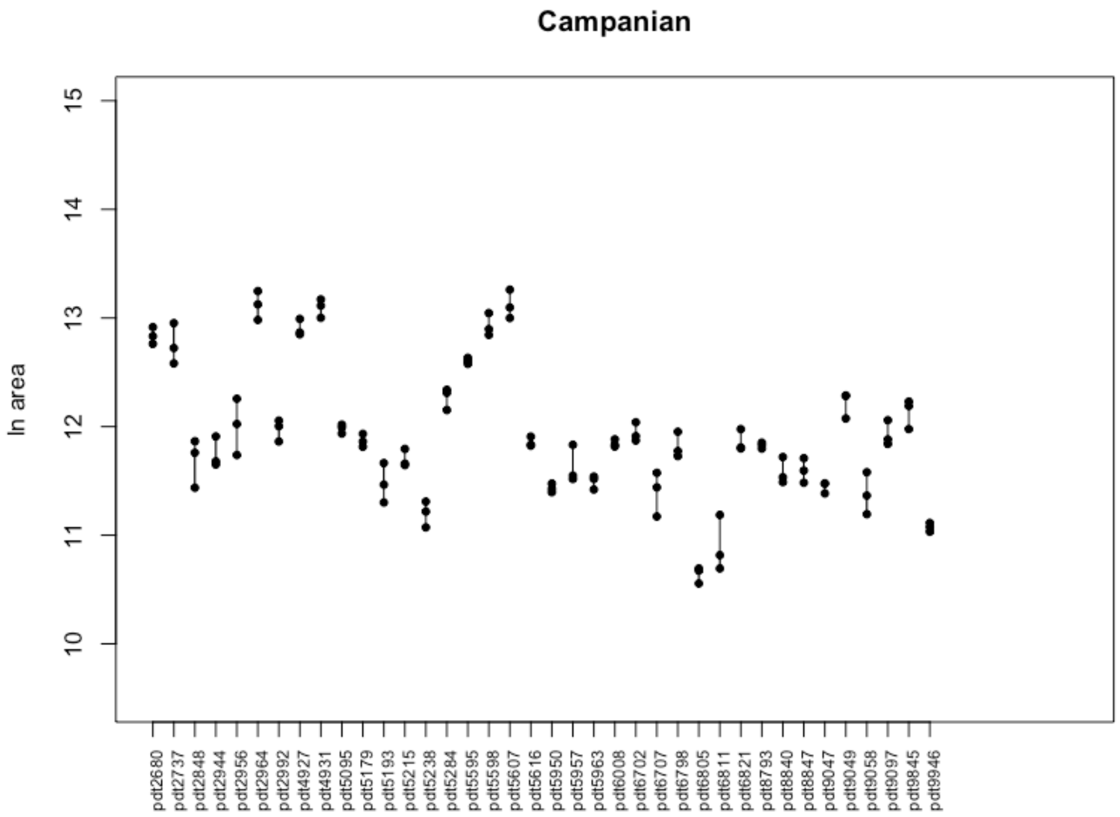
Campanian
ln area
10
11
12
13
14
15
pdt2680
pdt2737
pdt2848
pdt2944
pdt2956
pdt2964
pdt2992
pdt4927
pdt4931
pdt5095
pdt5179
pdt5193
pdt5215
pdt5238
pdt5284
pdt5595
pdt5598
pdt5607
pdt5616
pdt5950
pdt5957
pdt5963
pdt6008
pdt6702
pdt6707
pdt6798
pdt6805
pdt6811
pdt6821
pdt8793
pdt8840
pdt8847
pdt9047
pdt9049
pdt9058
pdt9097
pdt9845
pdt9946

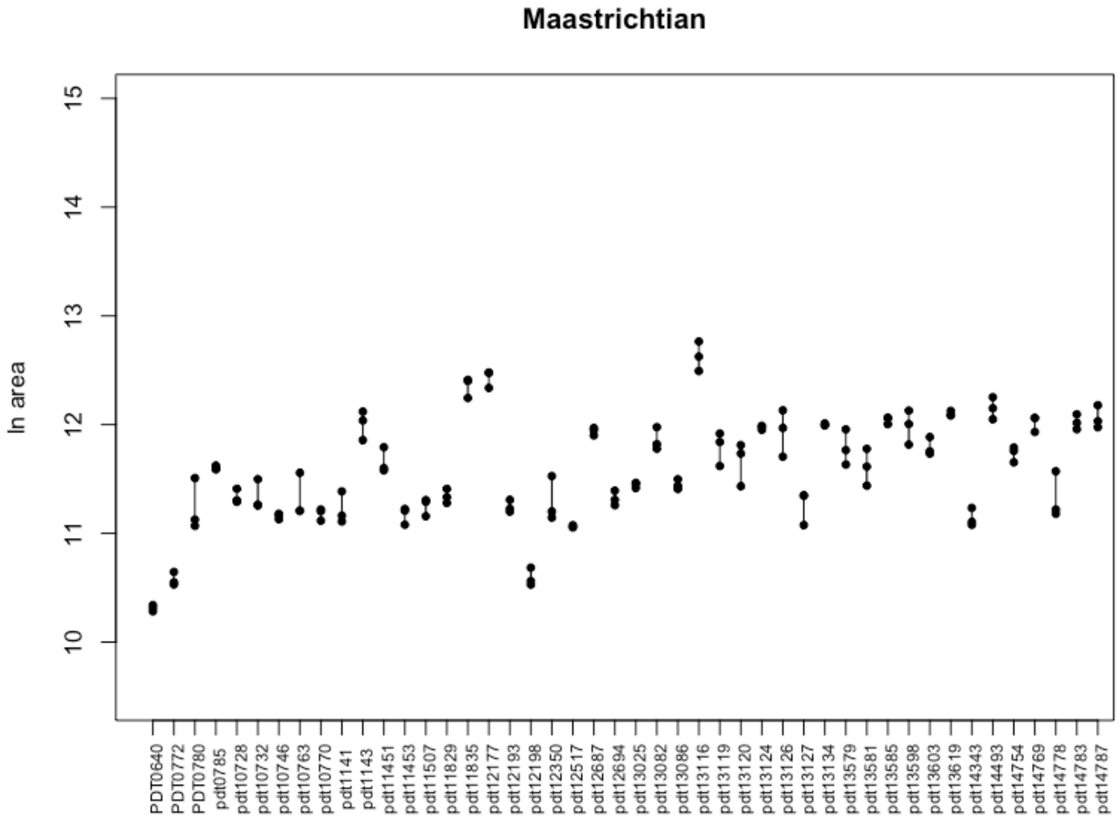
Maastrichtian
ln area
10
11
12
13
14
15
PDT0640
PDT0772
PDT0780
pdt0785
pdt10728
pdt10732
pdt10746
pdt10763
pdt10770
pdt1141
pdt1143
pdt11451
pdt11453
pdt11507
pdt11829
pdt11835
pdt12177
pdt12193
pdt12198
pdt12350
pdt12517
pdt12687
pdt12694
pdt13025
pdt13082
pdt13086
pdt13116
pdt13119
pdt13120
pdt13124
pdt13126
pdt13127
pdt13134
pdt13579
pdt13581
pdt13585
pdt13598
pdt13603
pdt13619
pdt14343
pdt14493
pdt14754
pdt14769
pdt14778
pdt14783
pdt14787

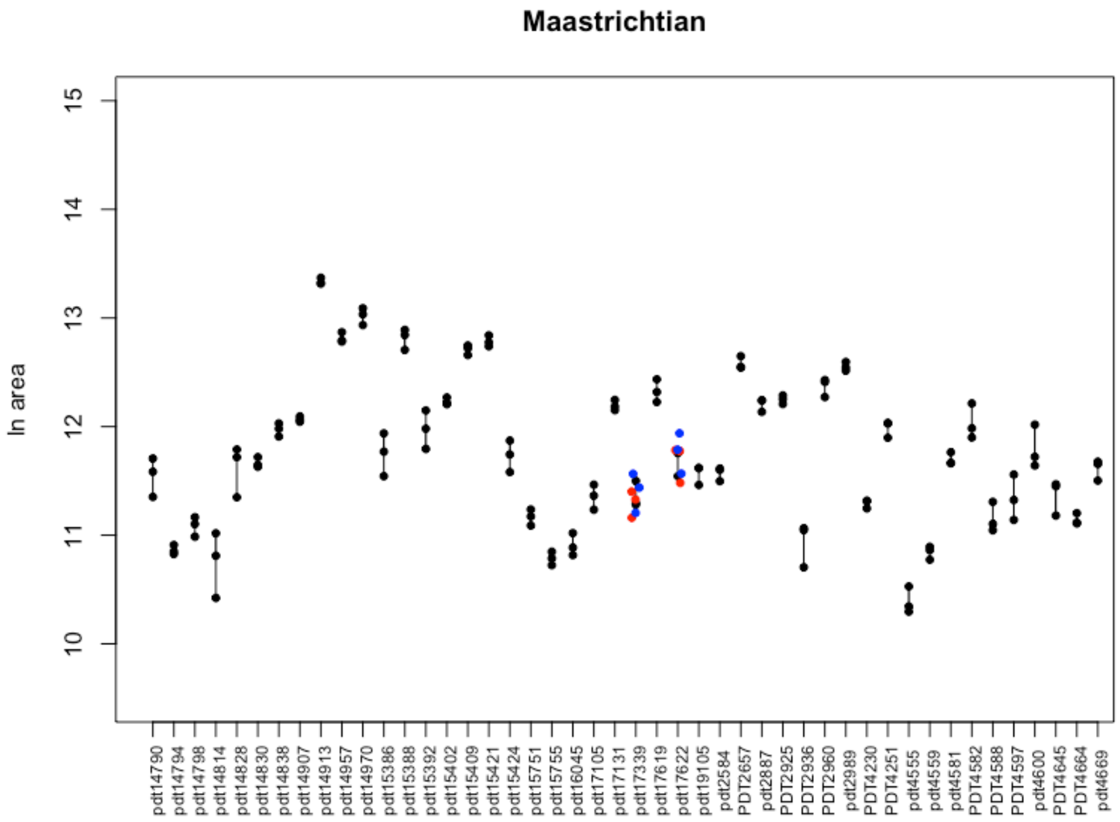
Maastrichtian
ln area
10
11
12
13
14
15
pdt14790
pdt14794
pdt14798
pdt14814
pdt14828
pdt14830
pdt14838
pdt14907
pdt14913
pdt14957
pdt14970
pdt15386
pdt15388
pdt15392
pdt15402
pdt15409
pdt15421
pdt15424
pdt15751
pdt15755
pdt16045
pdt17105
pdt17131
pdt17339
pdt17619
pdt17622
pdt19105
pdt2584
PDT2657
pdt2887
PDT2925
PDT2936
PDT2960
pdt2989
PDT4230
PDT4251
pdt4555
pdt4559
pdt4581
PDT4582
PDT4588
PDT4597
pdt4600
PDT4645
PDT4664
pdt4669

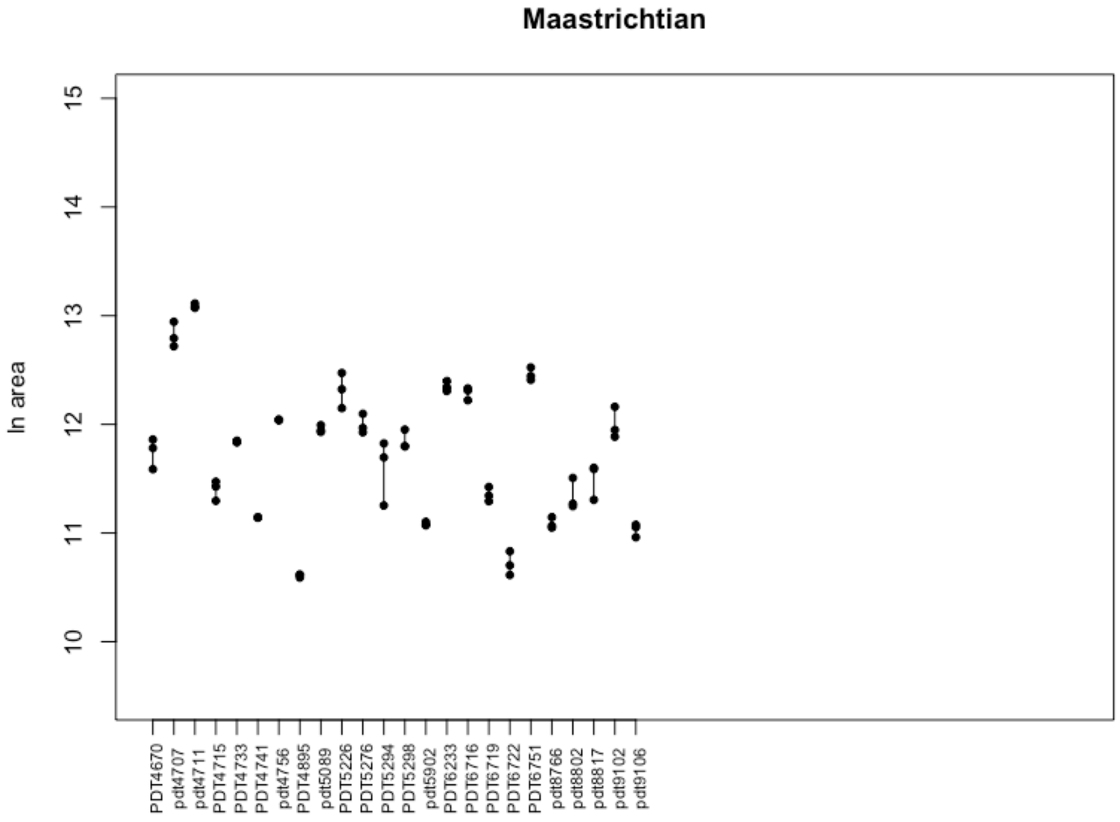
Maastrichtian
ln area
10
11
12
13
14
15
PDT4670
pdt4707
pdt4711
PDT4715
PDT4733
PDT4741
pdt4756
PDT4895
pdt5089
PDT5226
PDT5276
PDT5294
PDT5298
pdt5902
PDT6233
PDT6716
PDT6719
PDT6722
PDT6751
pdt8766
pdt8802
pdt8817
pdt9102
pdt9106

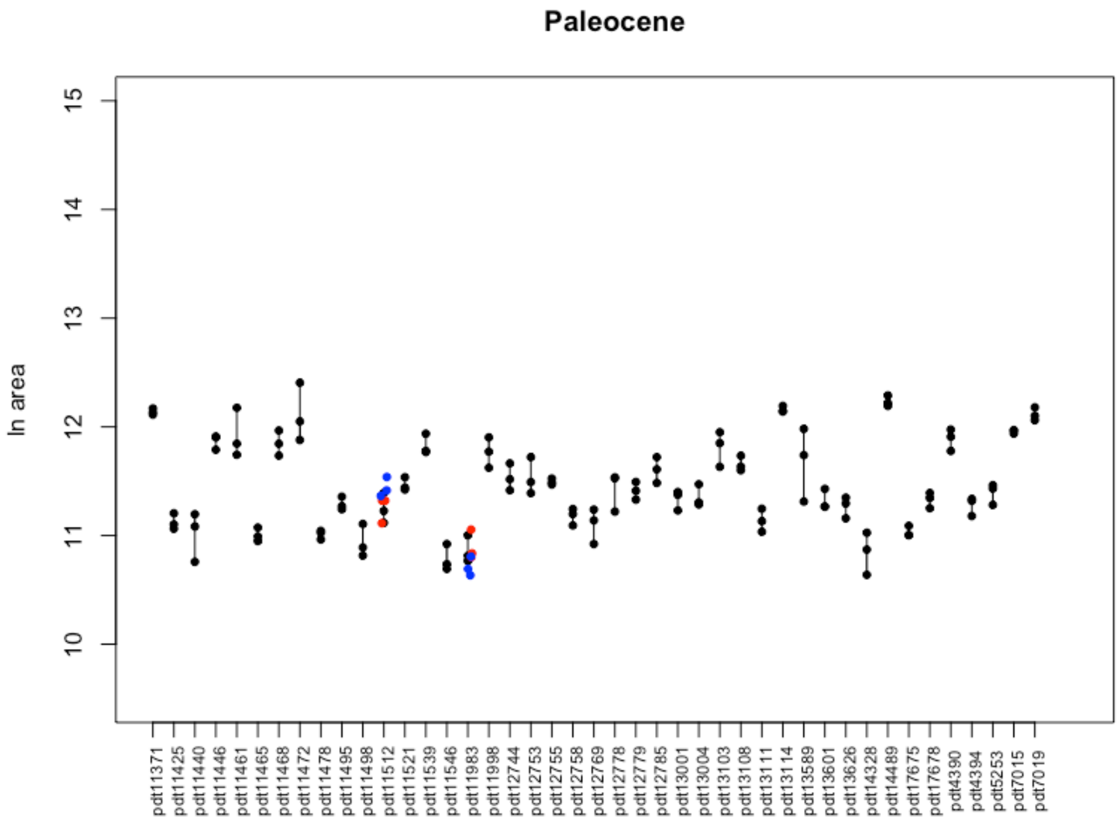
Paleocene
ln area
15
14
13
12
11
10
pdt11371
pdt11425
pdt11440
pdt11446
pdt11461
pdt11465
pdt11468
pdt11472
pdt11478
pdt11495
pdt11498
pdt11512
pdt11521
pdt11539
pdt11546
pdt11983
pdt11998
pdt12744
pdt12753
pdt12755
pdt12758
pdt12769
pdt12778
pdt12779
pdt12785
pdt13001
pdt13004
pdt13103
pdt13108
pdt13111
pdt13114
pdt13589
pdt13601
pdt13626
pdt14328
pdt14489
pdt17675
pdt17678
pdt4390
pdt4394
pdt5253
pdt7015
pdt7019

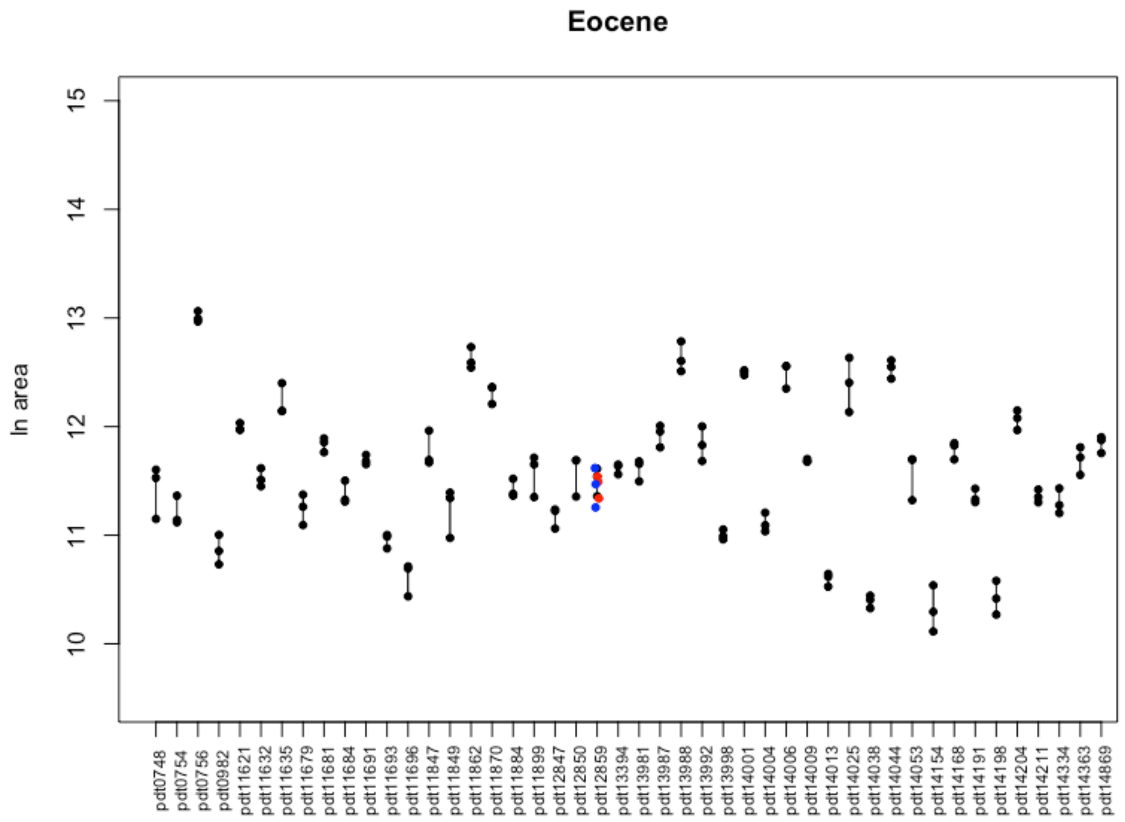
Eocene
ln area
15
14
13
12
11
10
pdt0748
pdt0754
pdt0756
pdt0982
pdt11621
pdt11632
pdt11635
pdt11679
pdt11681
pdt11684
pdt11691
pdt11693
pdt11696
pdt11847
pdt11849
pdt11862
pdt11870
pdt11884
pdt11899
pdt12847
pdt12850
pdt12859
pdt13394
pdt13981
pdt13987
pdt13988
pdt13992
pdt13998
pdt14001
pdt14004
pdt14006
pdt14009
pdt14013
pdt14025
pdt14038
pdt14044
pdt14053
pdt14154
pdt14168
pdt14191
pdt14198
pdt14204
pdt14211
pdt14334
pdt14363
pdt14869

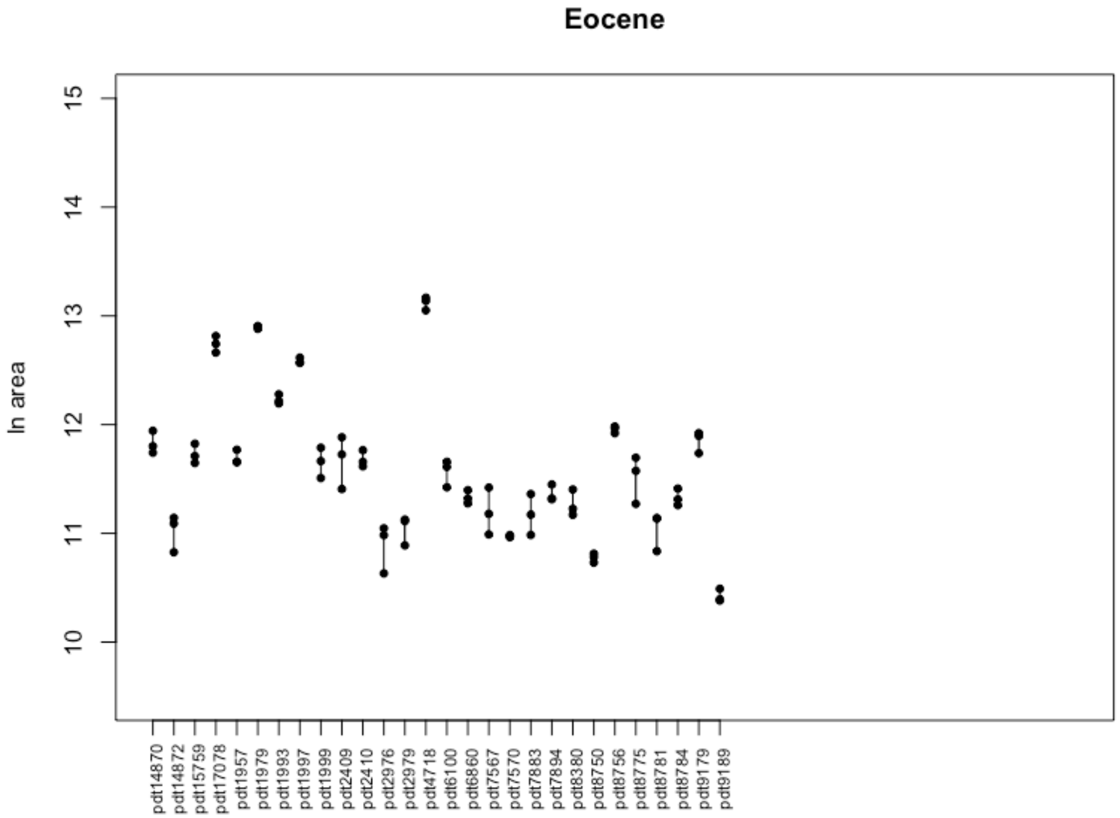
Eocene
ln area
15
14
13
12
11
10
pdt14870
pdt14872
pdt15759
pdt17078
pdt1957
pdt1979
pdt1993
pdt1997
pdt1999
pdt2409
pdt2410
pdt2976
pdt2979
pdt4718
pdt6100
pdt6860
pdt7567
pdt7570
pdt7883
pdt7894
pdt8380
pdt8750
pdt8756
pdt8775
pdt8781
pdt8784
pdt9179
pdt9189

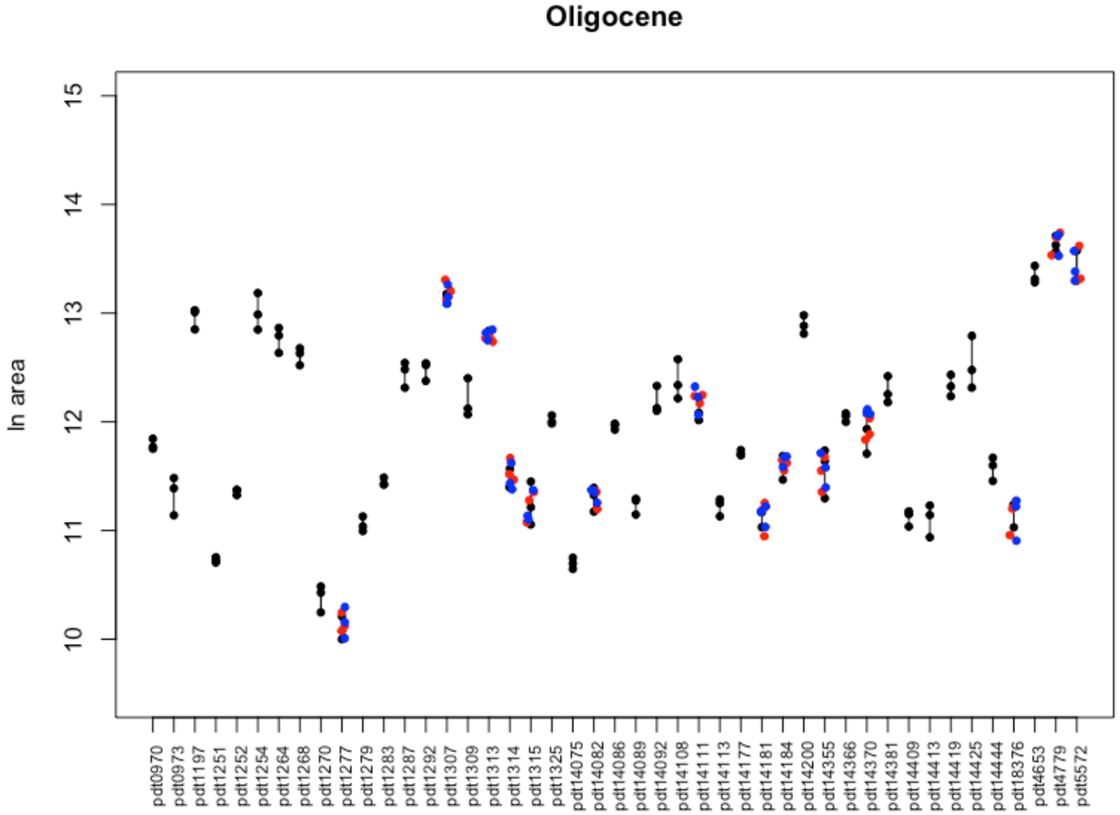
Oligocene
ln area
10
11
12
13
14
15
pdt0970
pdt0973
pdt1197
pdt1251
pdt1252
pdt1254
pdt1264
pdt1268
pdt1270
pdt1277
pdt1279
pdt1283
pdt1287
pdt1292
pdt1307
pdt1309
pdt1313
pdt1314
pdt1315
pdt1325
pdt14075
pdt14082
pdt14086
pdt14089
pdt14092
pdt14108
pdt14111
pdt14113
pdt14177
pdt14181
pdt14184
pdt14200
pdt14355
pdt14366
pdt14370
pdt14381
pdt14409
pdt14413
pdt14419
pdt14425
pdt14444
pdt18376
pdt4653
pdt4779
pdt5572

42

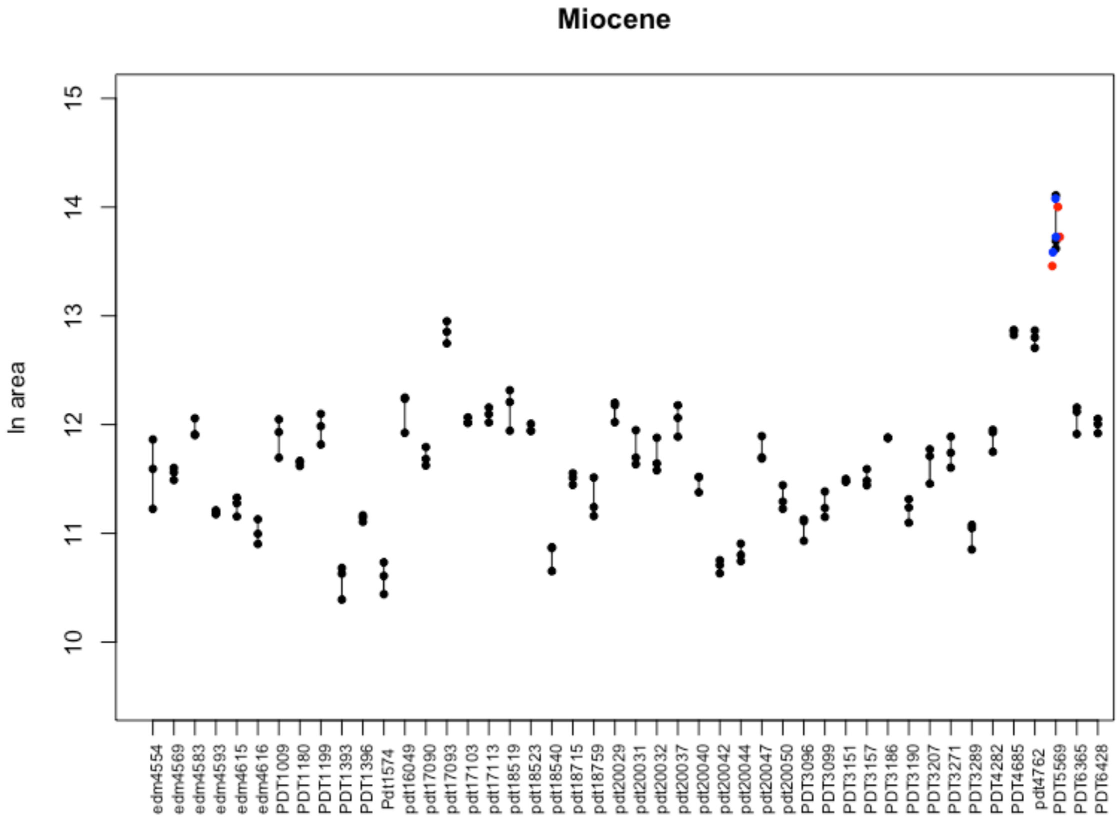
Miocene
ln area
10
11
12
13
14
15
edm4554
edm4569
edm4583
edm4593
edm4615
edm4616
PDT1009
PDT1180
PDT1199
PDT1393
PDT1396
Pdt1574
pdt16049
pdt17090
pdt17093
pdt17103
pdt17113
pdt18519
pdt18523
pdt18540
pdt18715
pdt18759
pdt20029
pdt20031
pdt20032
pdt20037
pdt20040
pdt20042
pdt20044
pdt20047
pdt20050
PDT3096
PDT3099
PDT3151
PDT3157
PDT3186
PDT3190
PDT3207
PDT3271
PDT3289
PDT4282
PDT4685
pdt4762
PDT5569
PDT6365
PDT6428

43

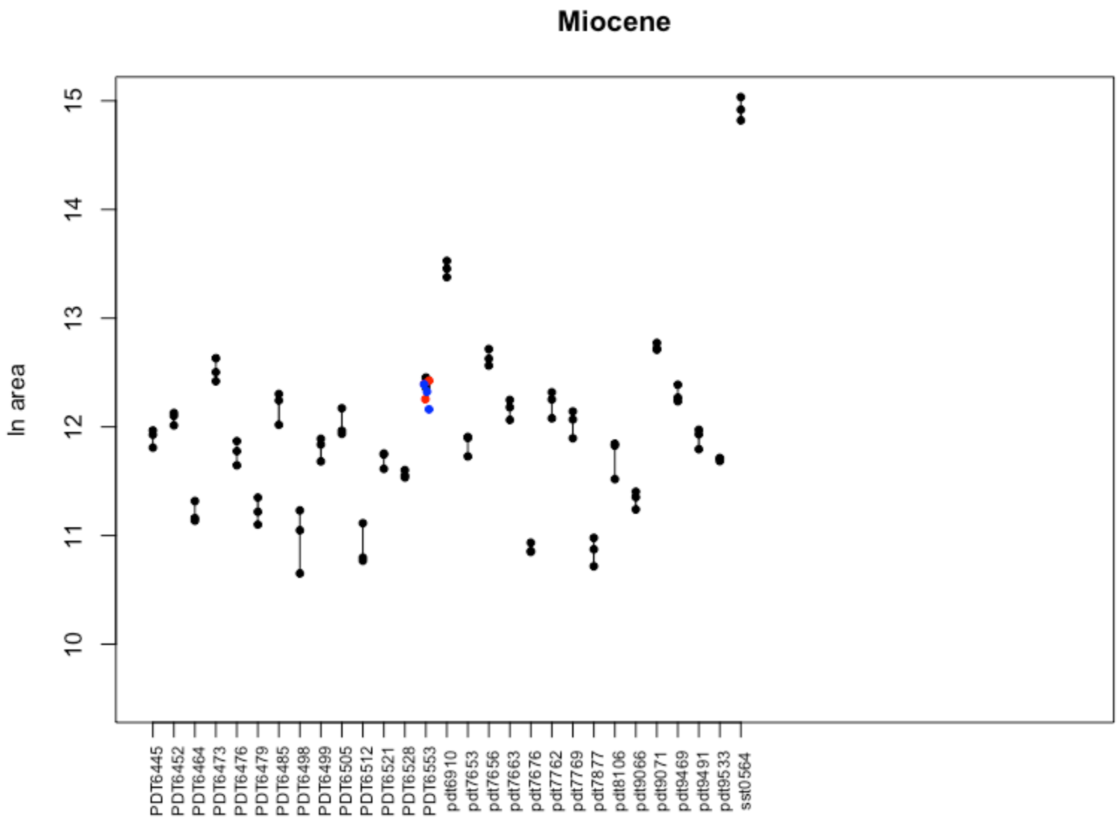
Miocene
ln area
15
14
13
12
11
10
PDT6445
PDT6452
PDT6464
PDT6473
PDT6476
PDT6479
PDT6485
PDT6498
PDT6499
PDT6505
PDT6512
PDT6521
PDT6528
PDT6553
pdt6910
pdt7653
pdt7656
pdt7663
pdt7676
pdt7762
pdt7769
pdt7877
pdt8106
pdt9066
pdt9071
pdt9469
pdt9491
pdt9533
sst0564

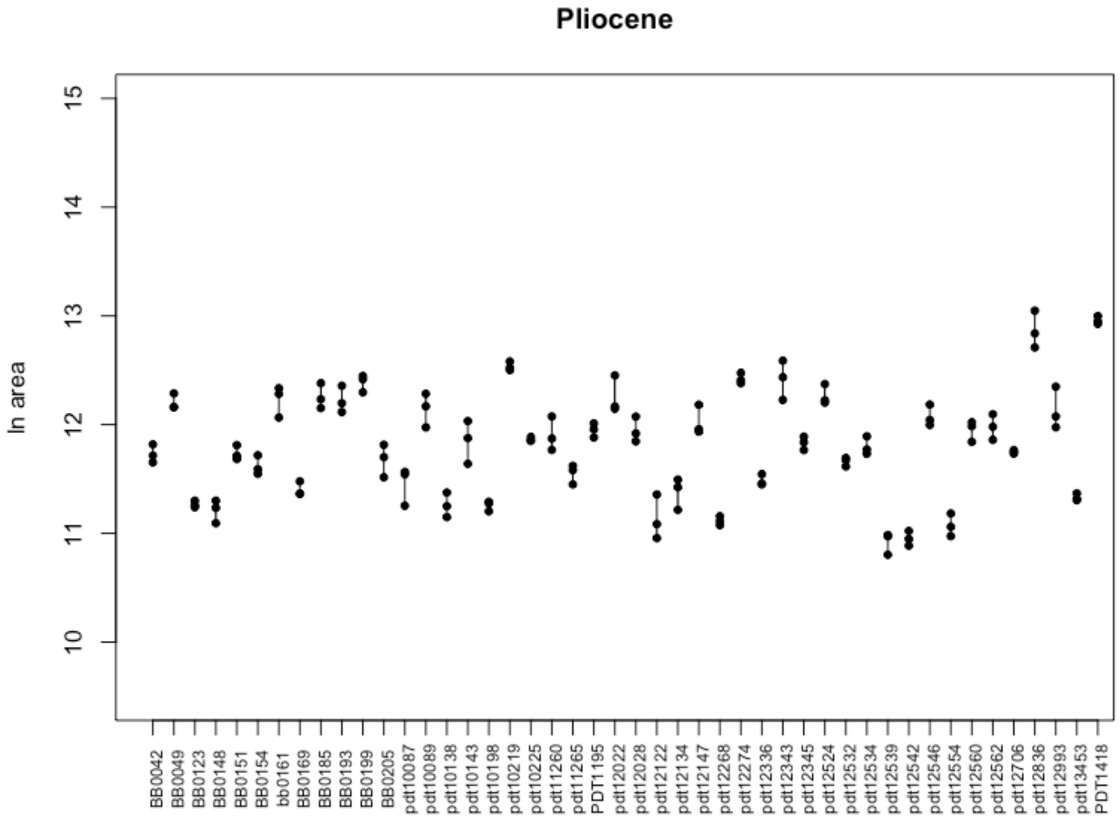
Pliocene
ln area
15
14
13
12
11
10
BB0042
BB0049
BB0123
BB0148
BB0151
BB0154
bb0161
BB0169
BB0185
BB0193
BB0199
BB0205
pdt10087
pdt10089
pdt10138
pdt10143
pdt10198
pdt10219
pdt10225
pdt11260
pdt11265
PDT1195
pdt12022
pdt12028
pdt12122
pdt12134
pdt12147
pdt12268
pdt12274
pdt12336
pdt12343
pdt12345
pdt12524
pdt12532
pdt12534
pdt12539
pdt12542
pdt12546
pdt12554
pdt12560
pdt12562
pdt12706
pdt12836
pdt12993
pdt13453
PDT1418

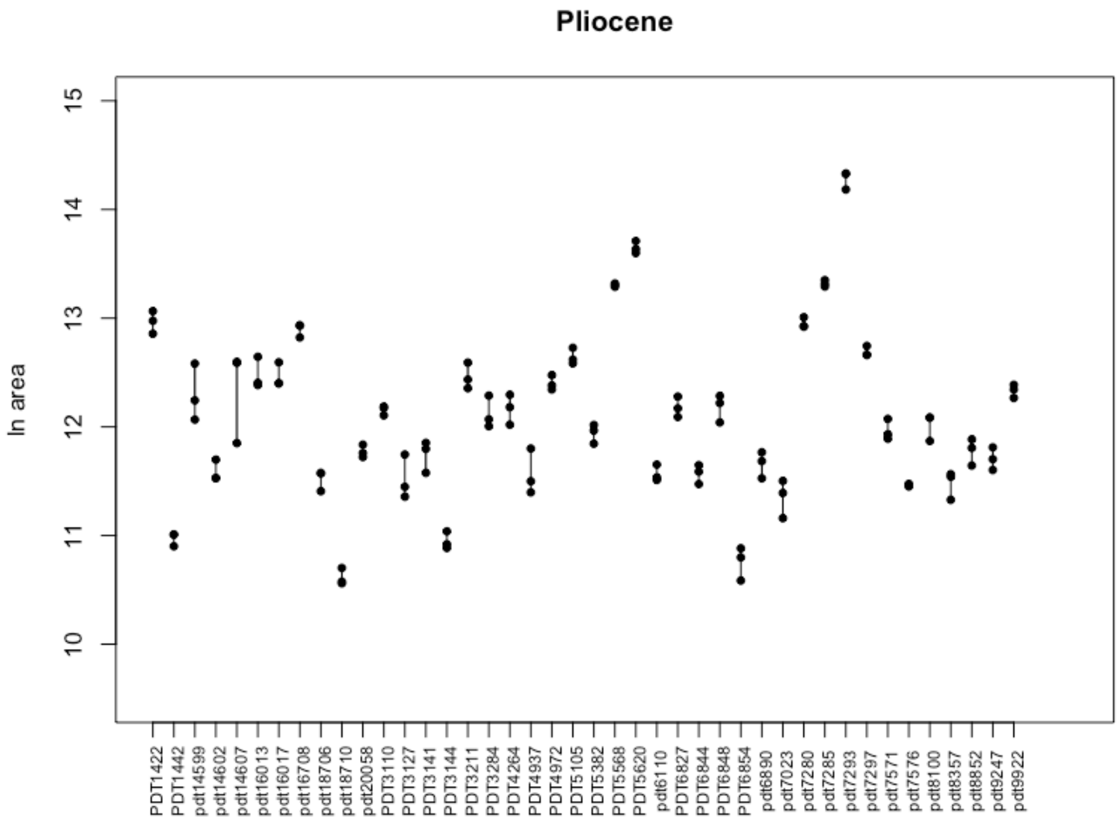
Pliocene
ln area
10
11
12
13
14
15
PDT1422
PDT1442
pdt14599
pdt14602
pdt14607
pdt16013
pdt16017
pdt16708
pdt18706
pdt18710
pdt20058
PDT3110
PDT3127
PDT3141
PDT3144
PDT3211
PDT3284
PDT4264
PDT4937
PDT4972
PDT5105
PDT5382
PDT5568
PDT5620
pdt6110
PDT6827
PDT6844
PDT6848
PDT6854
pdt6890
pdt7023
pdt7280
pdt7285
pdt7293
pdt7297
pdt7571
pdt7576
pdt8100
pdt8357
pdt8852
pdt9247
pdt9922

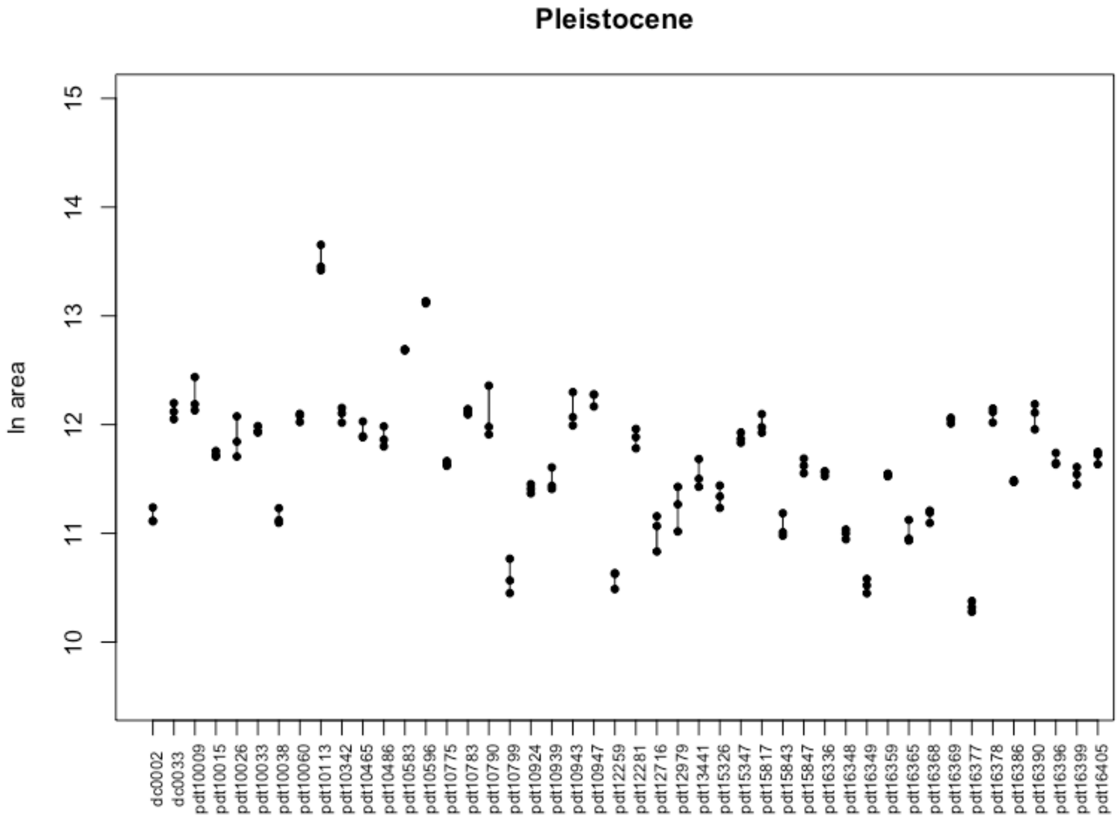
Pleistocene
ln area
10
11
12
13
14
15
dc0002
dc0033
pdt10009
pdt10015
pdt10026
pdt10033
pdt10038
pdt10060
pdt10113
pdt10342
pdt10465
pdt10486
pdt10583
pdt10596
pdt10775
pdt10783
pdt10790
pdt10799
pdt10924
pdt10939
pdt10943
pdt10947
pdt12259
pdt12281
pdt12716
pdt12979
pdt13441
pdt15326
pdt15347
pdt15817
pdt15843
pdt15847
pdt16336
pdt16348
pdt16349
pdt16359
pdt16365
pdt16368
pdt16369
pdt16377
pdt16378
pdt16386
pdt16390
pdt16396
pdt16399
pdt16405

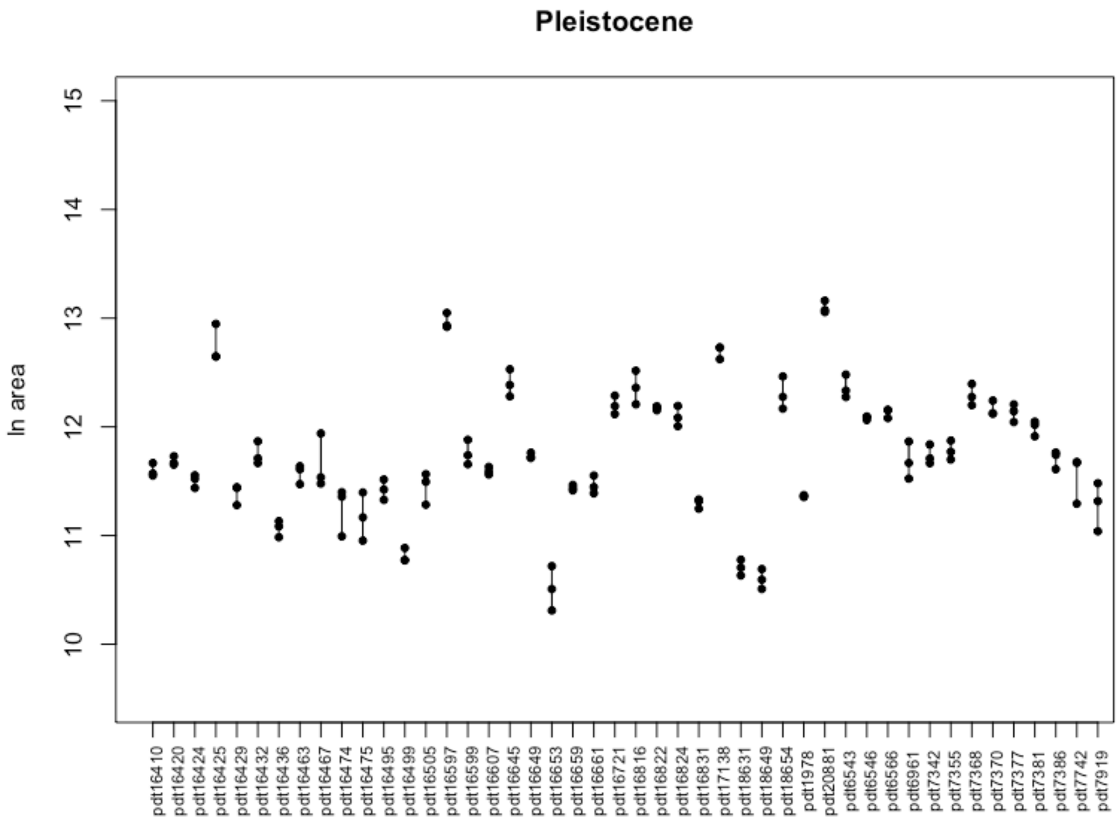
Pleistocene
ln area
15
14
13
12
11
10
pdt16410
pdt16420
pdt16424
pdt16425
pdt16429
pdt16432
pdt16436
pdt16463
pdt16467
pdt16474
pdt16475
pdt16495
pdt16499
pdt16505
pdt16597
pdt16599
pdt16607
pdt16645
pdt16649
pdt16653
pdt16659
pdt16661
pdt16721
pdt16816
pdt16822
pdt16824
pdt16831
pdt17138
pdt18631
pdt18649
pdt18654
pdt1978
pdt20881
pdt6543
pdt6546
pdt6566
pdt6961
pdt7342
pdt7355
pdt7368
pdt7370
pdt7377
pdt7381
pdt7386
pdt7742
pdt7919

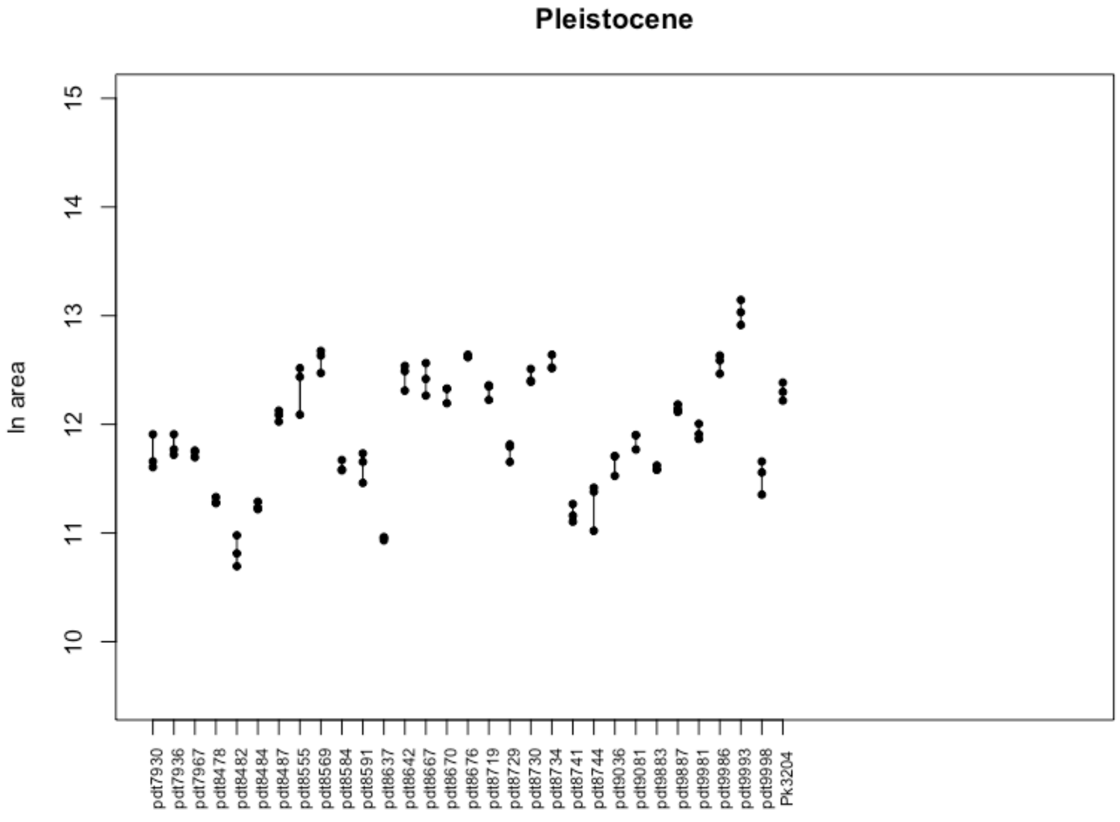
Pleistocene
ln area
15
14
13
12
11
10
pdt7930
pdt7936
pdt7967
pdt8478
pdt8482
pdt8484
pdt8487
pdt8555
pdt8569
pdt8584
pdt8591
pdt8637
pdt8642
pdt8667
pdt8670
pdt8676
pdt8719
pdt8729
pdt8730
pdt8734
pdt8741
pdt8744
pdt9036
pdt9081
pdt9883
pdt9887
pdt9981
pdt9986
pdt9993
pdt9998
Pk3204

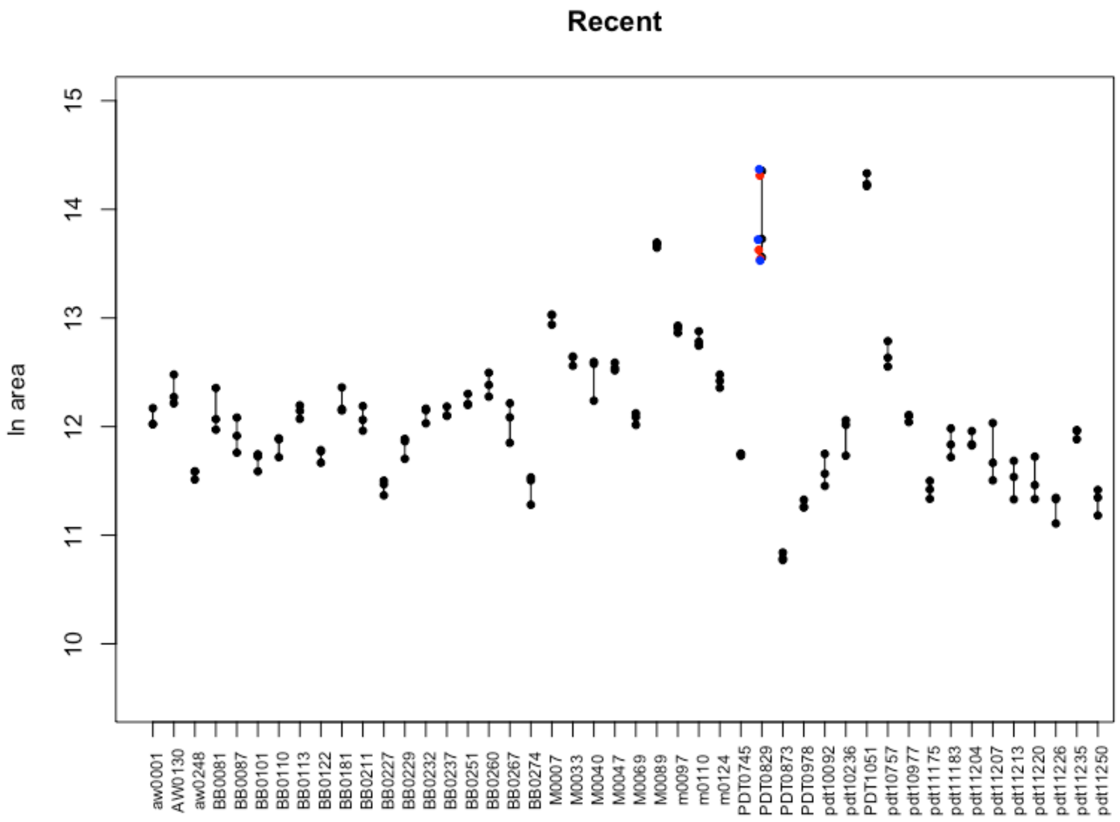
Recent
ln area
10
11
12
13
14
15
aw0001
AW0130
aw0248
BB0081
BB0087
BB0101
BB0110
BB0113
BB0122
BB0181
BB0211
BB0227
BB0229
BB0232
BB0237
BB0251
BB0260
BB0267
BB0274
M0007
M0033
M0040
M0047
M0069
M0089
m0097
m0110
m0124
PDT0745
PDT0829
PDT0873
PDT0978
pdt10092
pdt10236
PDT1051
pdt10757
pdt10977
pdt11175
pdt11183
pdt11204
pdt11207
pdt11213
pdt11220
pdt11226
pdt11235
pdt11250

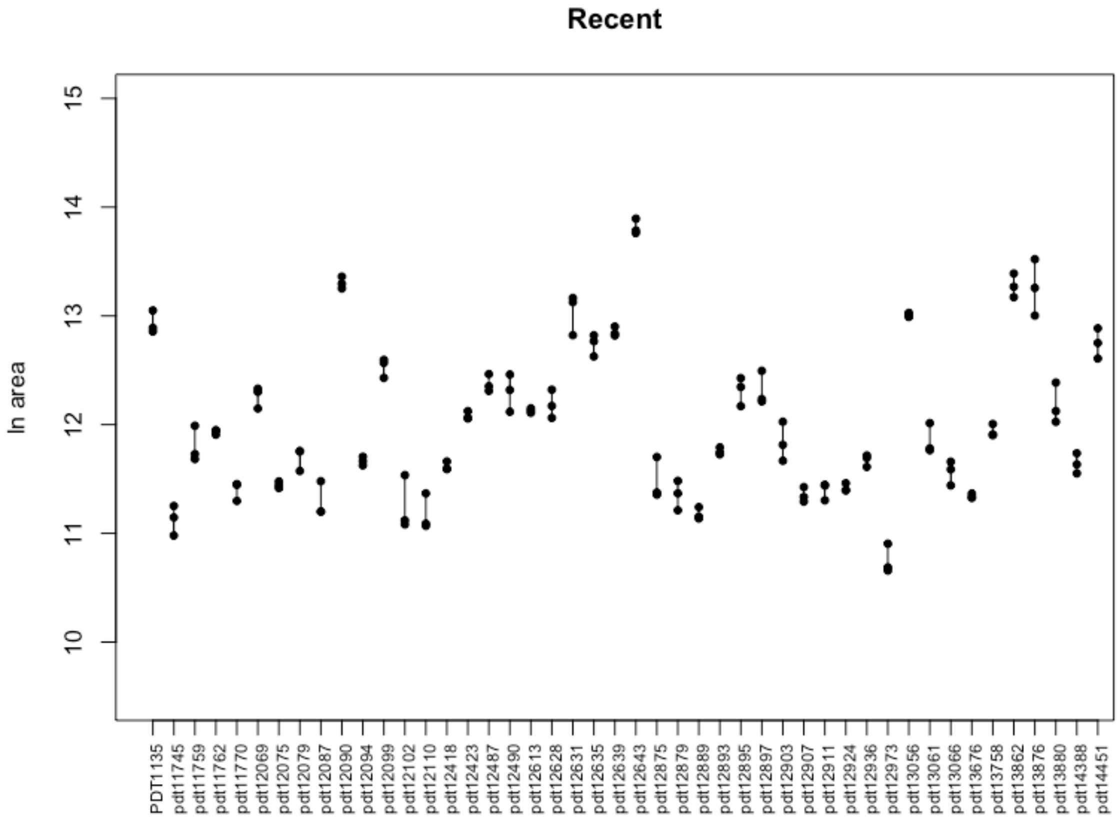
Recent
ln area
10
11
12
13
14
15
PDT1135
pdt11745
pdt11759
pdt11762
pdt11770
pdt12069
pdt12075
pdt12079
pdt12087
pdt12090
pdt12094
pdt12099
pdt12102
pdt12110
pdt12418
pdt12423
pdt12487
pdt12490
pdt12613
pdt12628
pdt12631
pdt12635
pdt12639
pdt12643
pdt12875
pdt12879
pdt12889
pdt12893
pdt12895
pdt12897
pdt12903
pdt12907
pdt12911
pdt12924
pdt12936
pdt12973
pdt13056
pdt13061
pdt13066
pdt13676
pdt13758
pdt13862
pdt13876
pdt13880
pdt14388
pdt14451

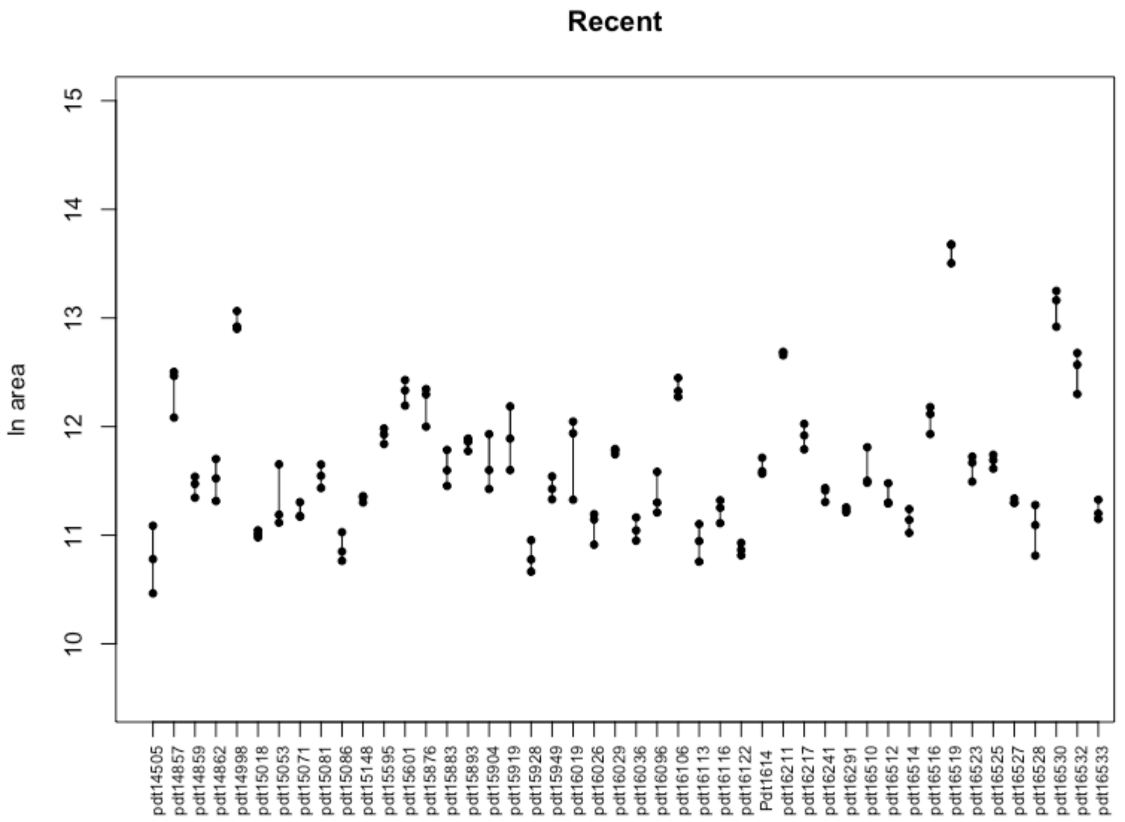
Recent
ln area
10
11
12
13
14
15
pdt14505
pdt14857
pdt14859
pdt14862
pdt14998
pdt15018
pdt15053
pdt15071
pdt15081
pdt15086
pdt15148
pdt15595
pdt15601
pdt15876
pdt15883
pdt15893
pdt15904
pdt15919
pdt15928
pdt15949
pdt16019
pdt16026
pdt16029
pdt16036
pdt16096
pdt16106
pdt16113
pdt16116
pdt16122
Pdt1614
pdt16211
pdt16217
pdt16241
pdt16291
pdt16510
pdt16512
pdt16514
pdt16516
pdt16519
pdt16523
pdt16525
pdt16527
pdt16528
pdt16530
pdt16532
pdt16533

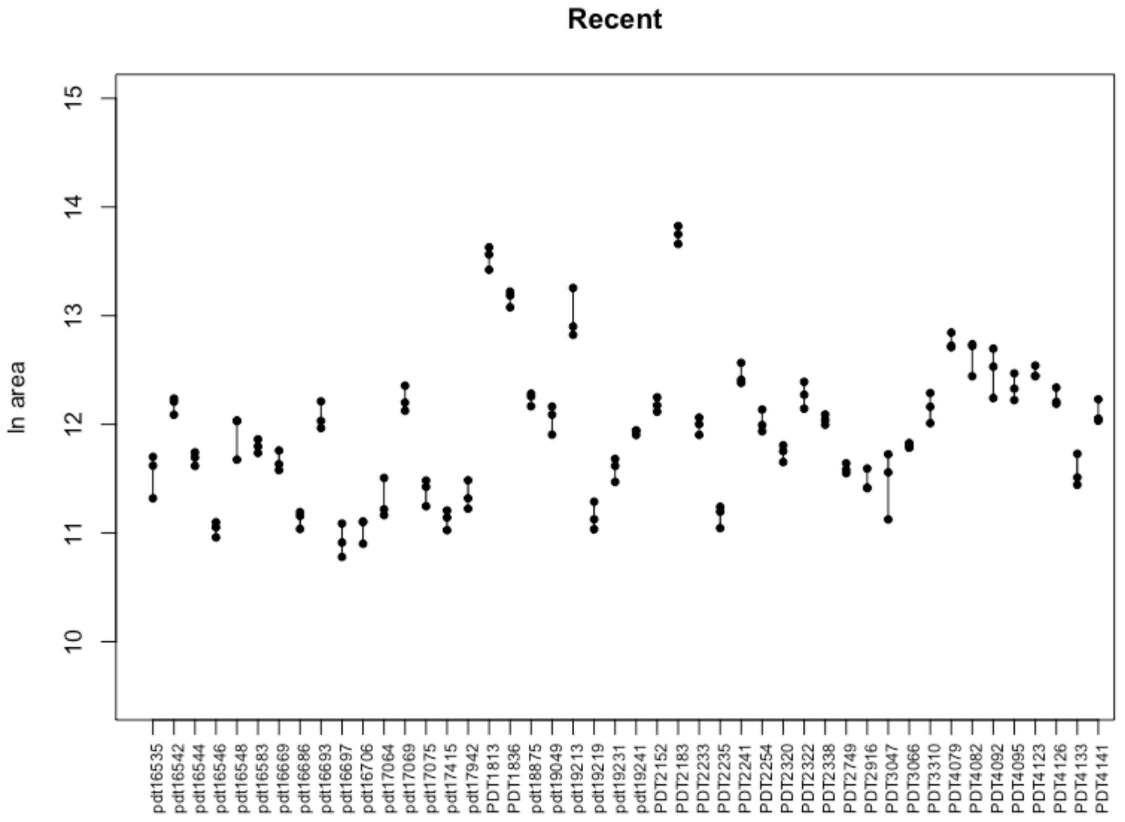
Recent
ln area
10
11
12
13
14
15
pdt16535
pdt16542
pdt16544
pdt16546
pdt16548
pdt16583
pdt16669
pdt16686
pdt16693
pdt16697
pdt16706
pdt17064
pdt17069
pdt17075
pdt17415
pdt17942
PDT1813
PDT1836
pdt18875
pdt19049
pdt19213
pdt19219
pdt19231
pdt19241
PDT2152
PDT2183
PDT2233
PDT2235
PDT2241
PDT2254
PDT2320
PDT2322
PDT2338
PDT2749
PDT2916
PDT3047
PDT3066
PDT3310
PDT4079
PDT4082
PDT4092
PDT4095
PDT4123
PDT4126
PDT4133
PDT4141

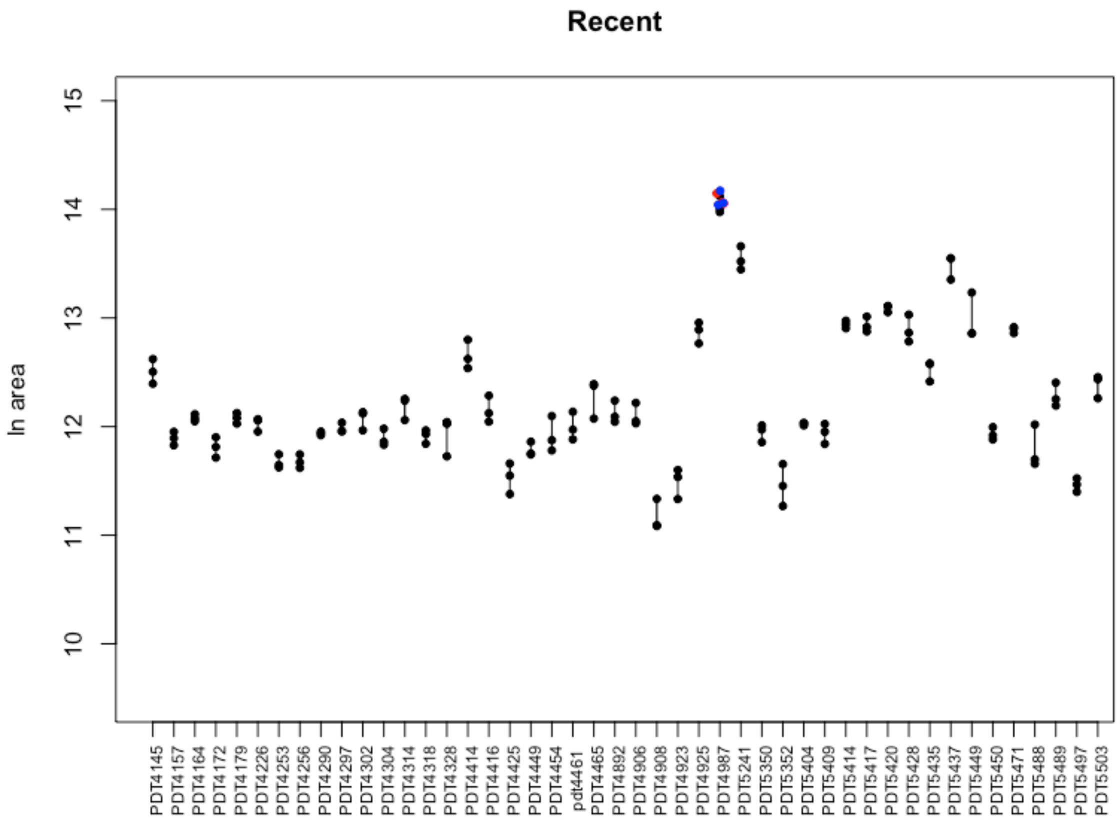
Recent
ln area
10
11
12
13
14
15
PDT4145
PDT4157
PDT4164
PDT4172
PDT4179
PDT4226
PDT4253
PDT4256
PDT4290
PDT4297
PDT4302
PDT4304
PDT4314
PDT4318
PDT4328
PDT4414
PDT4416
PDT4425
PDT4449
PDT4454
pdt4461
PDT4465
PDT4892
PDT4906
PDT4908
PDT4923
PDT4925
PDT4987
PDT5241
PDT5350
PDT5352
PDT5404
PDT5409
PDT5414
PDT5417
PDT5420
PDT5428
PDT5435
PDT5437
PDT5449
PDT5450
PDT5471
PDT5488
PDT5489
PDT5497
PDT5503

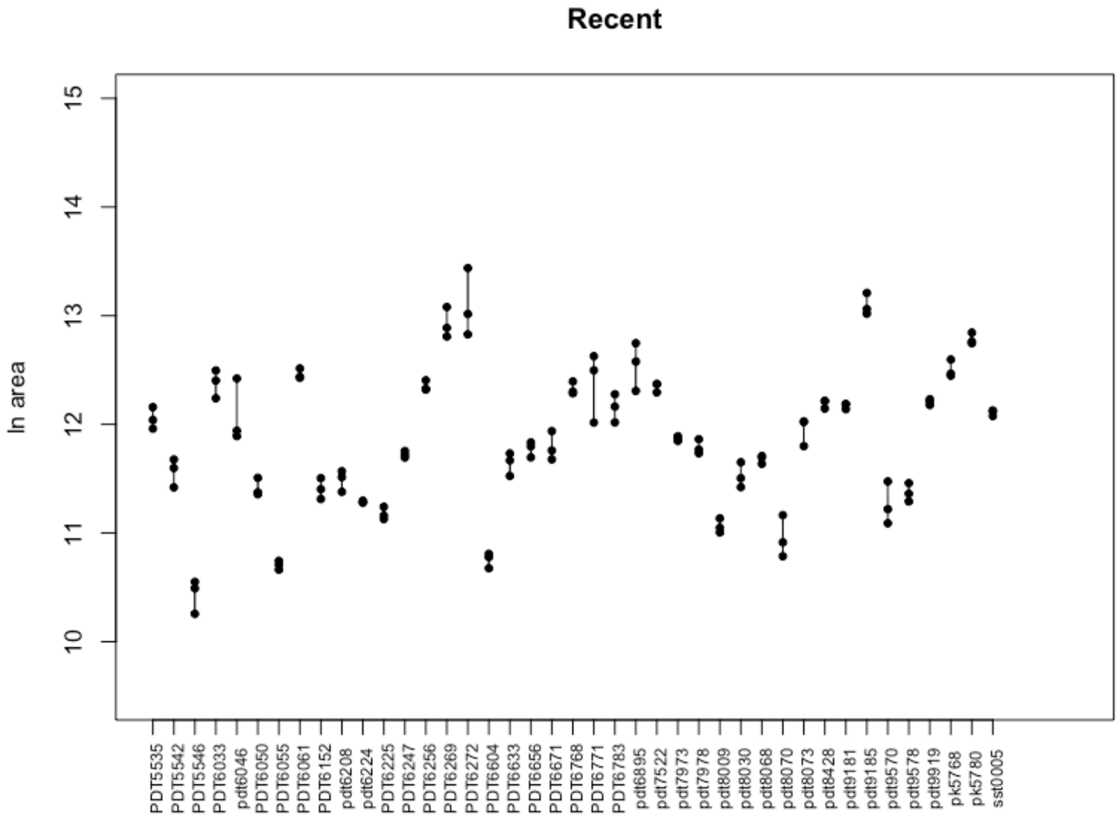
Recent
ln area
10
11
12
13
14
15
PDT5535
PDT5542
PDT5546
PDT6033
pdt6046
PDT6050
PDT6055
PDT6061
PDT6152
pdt6208
pdt6224
PDT6225
PDT6247
PDT6256
PDT6269
PDT6272
PDT6604
PDT6633
PDT6656
PDT6671
PDT6768
PDT6771
PDT6783
pdt6895
pdt7522
pdt7973
pdt7978
pdt8009
pdt8030
pdt8068
pdt8070
pdt8073
pdt8428
pdt9181
pdt9185
pdt9570
pdt9578
pdt9919
pk5768
pk5780
sst0005

**Fig. S2. Length, width, area relationships.** Each point represents a measurement of a zooid (natural logged in µm or µm$^2$) , from only the first measurement of any species (N = 3504). Zooid areas are square-rooted to reduce it to one dimension, except in the last panel. Red lines are fitted linear models and r.sq are adjusted r-squared values from the linear fit. Note that both length and width are reasonable proxies for area, but that length and width do not correlate well with each other. However, length times width is a good proxy for area (adjusted r-square = 0.958).

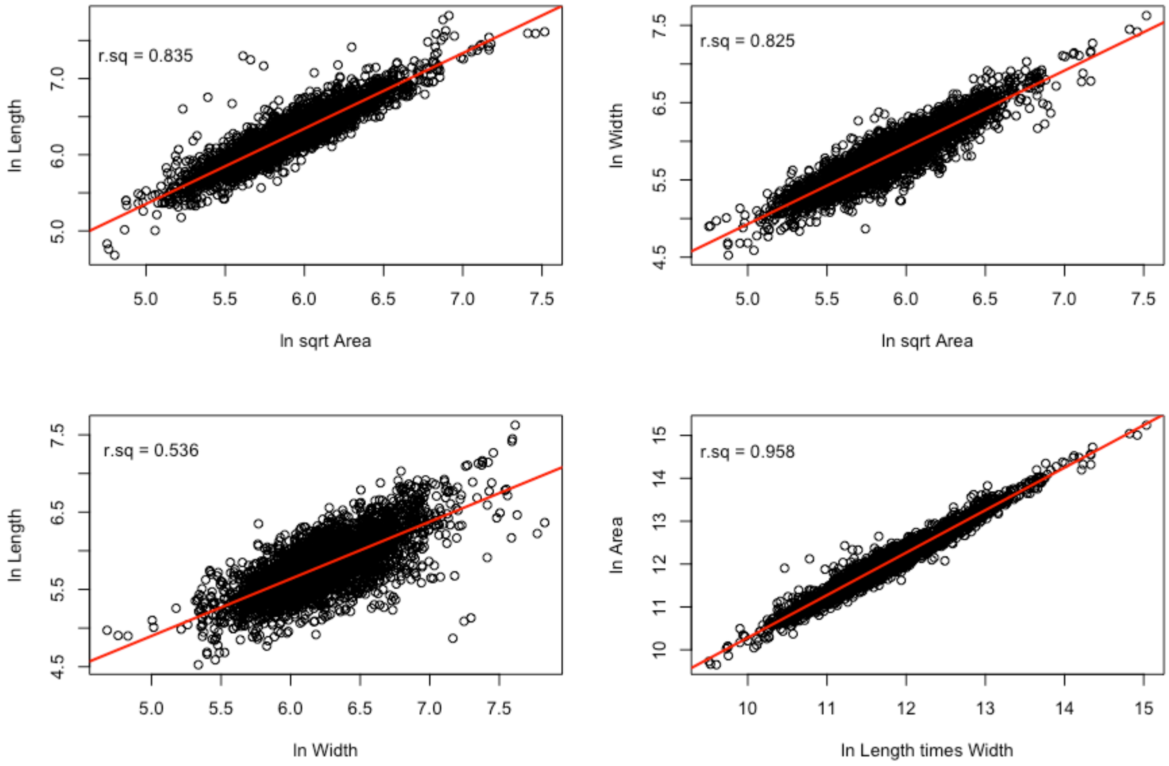

**Fig. S3. Distribution of ln zooid area.** A histogram showing the species means of all 1168 species. The grand median is 11.74 (plotted in blue) while the grand mean is 11.76 µ$m^2$.

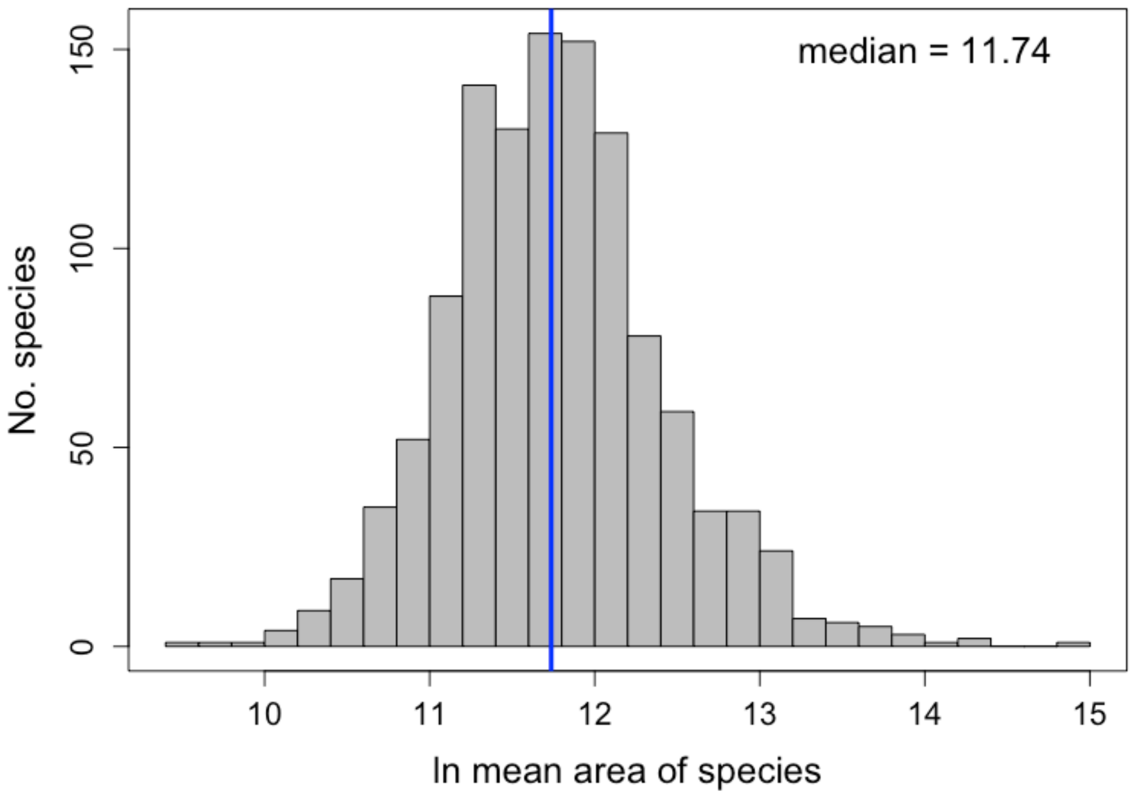

**Fig. S4. Autozooid area over time**. A. Boxplots of mean ln area μm$^2$ of cheilostome species sampled within international geological stages. B. The same data for 11 million year (myr) time intervals equivalent to one of the defaults used by the Paleobiology Database. As in main text Fig. 2, boxes show interquartile ranges (IQR) while whiskers extend to 1.5 times IQR. Grey open circles are individual species means. Numbers above the boxes are the number of species represented in the given time interval. The red lines are the grand median while the blue lines are the grand means.

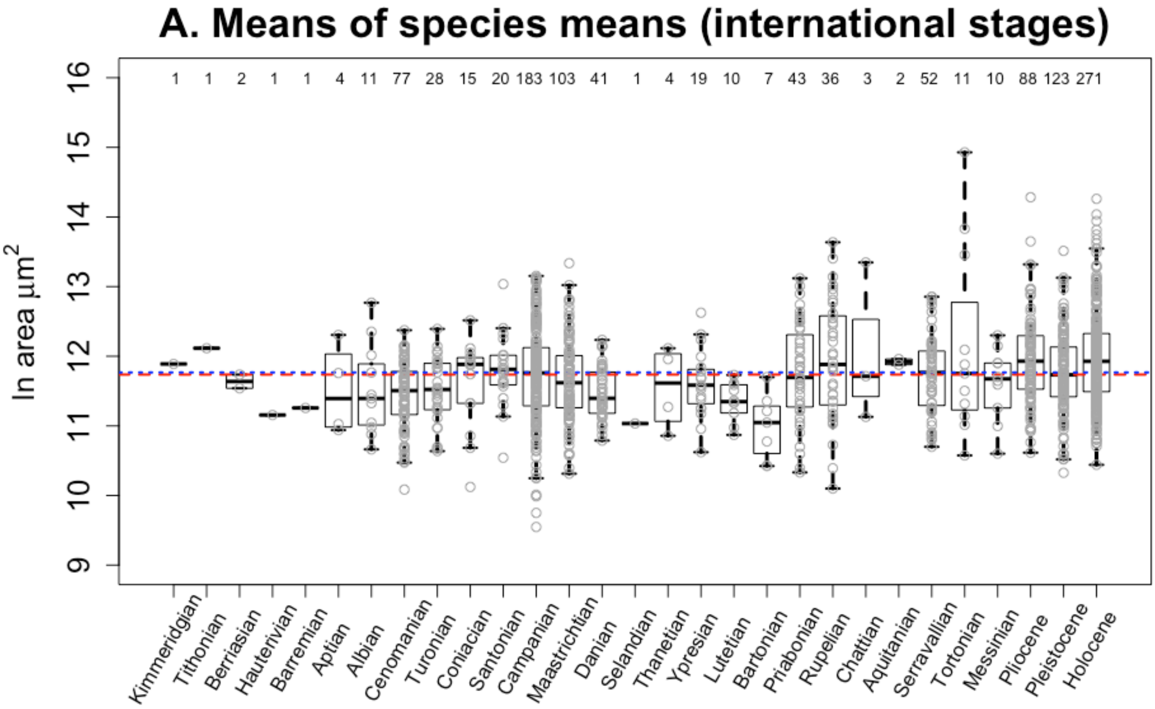


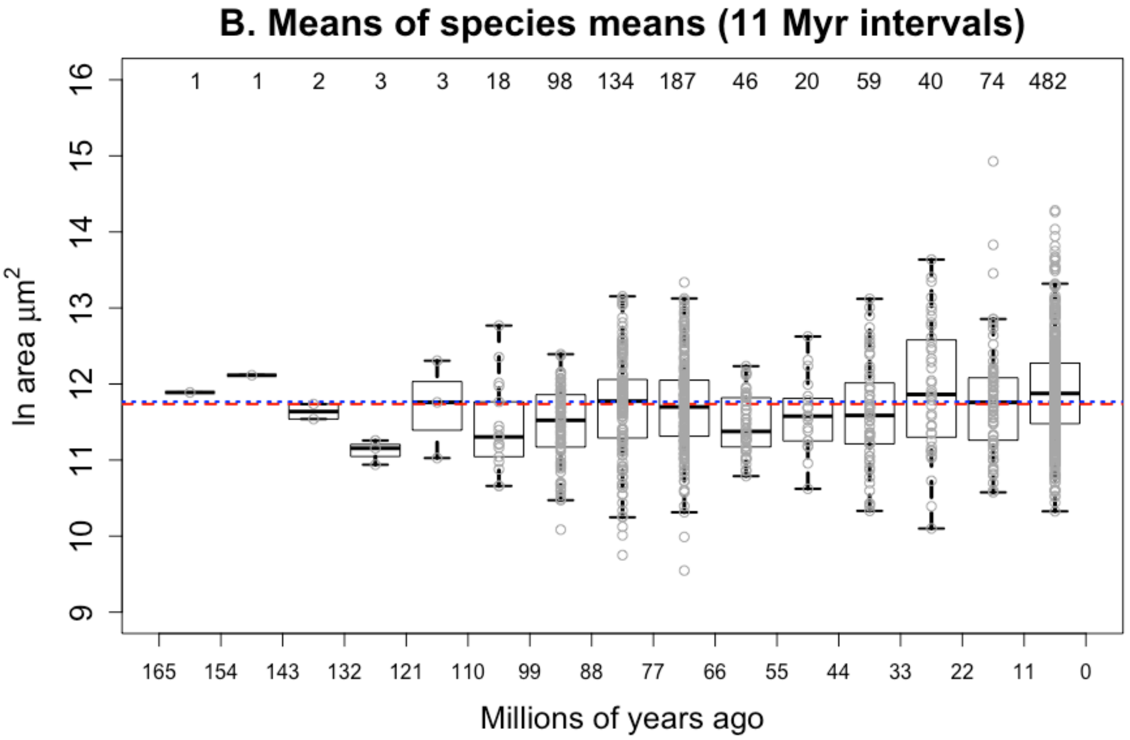

**Table S1. Phenotypic change model weights.** The first column indicates the models used. The column '10-million-year bins' shows model weights for data where the ln zooid area data are averaged in evenly spaced 10-million-year bins (data as in main text Fig. 2). The column 'Stages bins' shows model weights for data where the ln zooid area are averaged in international geological stages (as in Fig. S4A). The column '11-million year bins' shows model weights for data where the ln zooid area data are averaged in evenly spaced 11-million-year bins (data as in Fig. S4B). The column 'Runs' shows means (and standard deviations in parentheses) of 1000 model weights when ages of species are drawn from the duration of the geological stage they were found in (see main text). The best models are in bold.

| Models | 10-million-year bins | International stages | 11-million-year bins | Runs |
|---|---|---|---|---|
| Unbiased Random Walk (URW) | 0.090 | 0.003 | 0.193 | **0.342** (0.169) |
| Generalized Random Walk (GRW) | 0.031 | 0.001 | 0.067 | 0.119 (0.575) |
| Stasis | **0.571** | **0.812** | **0.712** | 0.249 (0.204) |
| Strict Stasis (SS) | 0 | 0 | 0 | 0 (0) |
| Ornstein Uhlenbeck model (OU) | 0.309 | 0.185 | 0.028 | 0.288 (0.233) |

**Fig. S5. Average probabilities of larger descendants within genera.** Each point (jittered to show overlapping points) plots the average binomial probability of having larger descendants in putative AD pairs in a genus, versus the number of species available for comparisons within that genus. Two of the most data-rich genera are labeled.

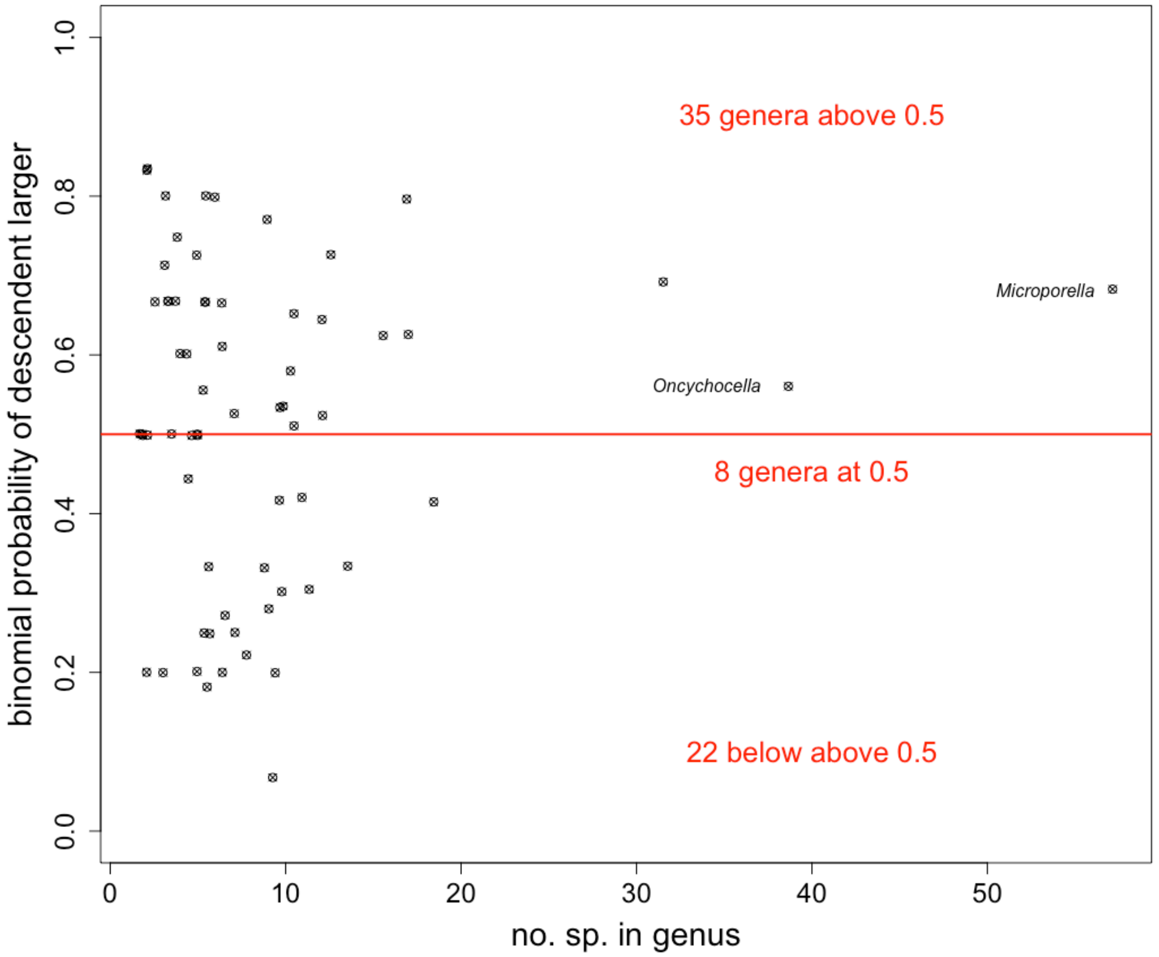

**Table S2 A comparison of basic random effects models.** Comparing four different random effects models indicated in the first column indicated using R notation. The columns are degree of difference (df), Akaike Information Criteria (AIC), followed by estimates for the fixed effect of log zooid area and standard error in parentheses, then random effect variances for species, repeat measurements in species, time (geologic stages) and residuals. Note that the third model includes time as fixed effects (hence the cell for random effects is empty). The df is 13 rather than 19 for the third model because some time intervals have no repeat measurements and are hence dropped from the analyses. Note that these analyses is based only on the colonies that have repeat measurements. The bold model is the best but the model with double asterisks is what will be used (see Table S3).

| Model | df | AIC | Fixed (area) | Random (species) | Random (repeats) | Random (Time) | Residuals |
|---|---|---|---|---|---|---|---|
| **~ (1\|species/repeat)** | 5 | -758 | 11.72 (0.10) | 0.708 | 0.016 | NA | 0.0041 |
| ~ (1 \| species)** | 3 | -365 | 11.72 (0.10) | 0.712 | NA | NA | 0.0163 |
| ~ Time + (1 \| species ) | 13 | -381 | 11.46 (0.17) | 0.415 | NA | | 0.0163 |
| ~ (1 \| Time) + (1 \| species ) | 4 | -368 | 11.81 (0.23) | 0.509 | NA | 0.419 | 0.0163 |

**Table S3 A comparison of mixed models including paleolatitude and growth forms.** Comparing five different random effects are indicated using R notation (first column). The five rows under "two colony forms" include only the most common forms, namely encrusting sheet (N= 854) and erect colonies (N = 249). The five rows under "four colony forms" include also encrusting runners (N = 31) and free-living colonies (N = 33). The columns are degree of difference (df), Akaike Information Criteria (AIC). Note that this analysis is based only all colonies that can be assigned to a latitude. The bold model is the best one in each case, as also confirmed by an Anova analysis.

| Model | df | AIC |
|---|---|---|
| Two colony forms | | |
| ~ (1 \| Time) +(1 \| species) | 4 | 291 |
| ~ Form+(1 \| Time) +( (1 \| species) | 5 | 291 |
| **~PaleoLat+(1 \| Time) +( (1 \| species)**[§,+] | 5 | 258 |
| ~PaleoLat +Form+(1 \| Time) +( (1 \| species)[§] | 6 | 244 |
| ~PaleoLat *Form+(1 \| Time) +( (1 \| species)[§] | 6 | 246 |
| Four colony forms | | |
| ~ (1 \| Time) +(1 \| species) | 4 | 328 |
| ~ Form+(1 \| Time) +( (1 \| species) | 7 | 311 |
| ~PaleoLat+(1 \| Time) +( (1 \| species) | 5 | 272 |
| **~PaleoLat +Form+(1 \| Time) +( (1 \| species)*** | 8 | 264 |
| ~PaleoLat *Form+(1 \| Time) +( (1 \| species) | 11 | 267 |

[§]These three models have different slightly different AICs but are not significantly different in a anova test and hence the simplest model has been highlighted as the overall best model.

[+]Estimates for the best model from two colony forms data: zooid size (fixed effect) = 11.20 (std error = 0.09); latitude (fixed effect) = 0.012 (std error = 0.002); time (random effect variance) = 0.035 (std. dev = 0.18); species (random effect variance) = 0.37 (std. dev = 0.61); residual variance = 0.015 (std. dev = 0.12)

[*] Estimates for the best model from four colony forms data: zooid size (fixed effect) = 11.20 (std error = 0.09); latitude (fixed effect) = 0.012 (std error = 0.002); Free-living (fixed effect) = -0.40 (std. error = 0.12); Runner (fixed effect) = -0.03 (std. error = 0.12); Encrusting Sheet (fixed effect) = 0.04 (std. error = 0.05) time (random effect variance) = 0.031 (std. dev = 0.18); species (random effect variance) = 0.37 (std. dev = 0.61); residual variance = 0.015 (std. dev = 0.12). Hence, encrusting sheets are have marginally larger zooids than erect colonies while runners and free-living forms have somewhat smaller zooids than erect colonies.

**Table S4. A comparison of mixed models for change in area from ancestor to descendent.** Comparing three different random effects models indicated in the first column indicated using R notation, where shift indicates the change in latitude and abs.time.diff is the absolute difference in time between the putative ancestor and descendent (using estimates to the closest million years). The columns are degree of difference (df), Akaike Information Criteria (AIC), followed by estimates for the fixed effects of change in log zooid area and standard error in parenthesis, shift in (paleo)latitude, and absolute time difference between ancestor and descendent followed by genus random effects and the residuals. The bold model is the best one.

| Model | df | AIC | Fixed (change in area) | Fixed (shift in latitude) | Fixed (time transpired) | Random (Genus) | Residual |
|---|---|---|---|---|---|---|---|
| **~(1\|genus)** | 3 | 8064 | -0.061 (0.029) | | | 0.04 | 0.44 |
| ~shift+(1\|genus) | 4 | 8065 | -0.060 (0.029) | 0.0005 (0.0006) | | 0.04 | 0.44 |
| ~shift+(1\|genus)+ abs.time.diff | 5 | 8067 | - 0.061 (0.030) | 0.0005 (0.0007) | 2.96e-06 (5.98e-04) | 0.04 | 0.44 |

**Fig. S6. Shift in latitude versus change in ln zooid size (from Ancestor to Descendent).** Each point represents a putative AD pair. The blue line is the null hypothesis, while the dotted red line is the estimated fit from the second model in Table S4. These are indistinguishable.

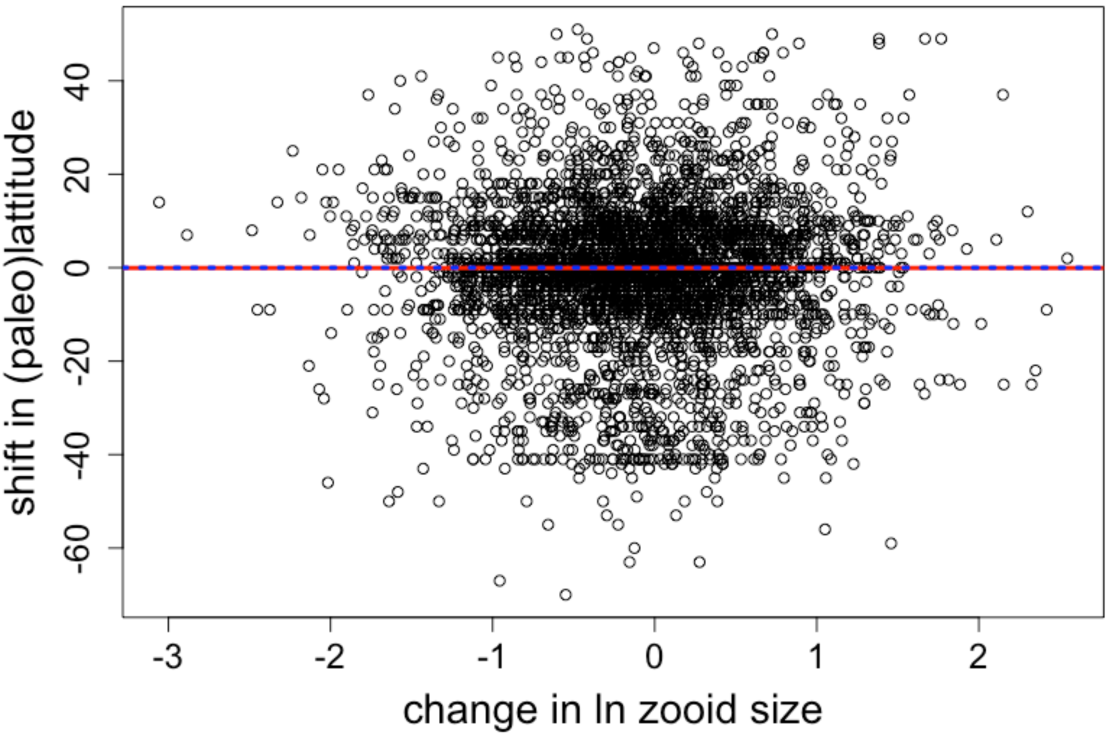

Supplement: Supplementary file 1 — Fig. S1. Zooid area measurements for each species. Fig. S2. Length, width, area relationships. Fig. S3. Distribution of ln zooid area. Fig. S4. Autozooid area over time. Fig. S5. Average probabilities of larger descendants within genera. Fig. S6. Shift in latitude versus change in ln zooid size (from ancestor to descendent). Table S1. Phenotypic change model weights. Table S2. A comparison of basic random effects models. Table S3. A comparison of mixed models including paleolatitude and growth forms. Table S4. A comparison of mixed models for change in area from ancestor to descendent. [file EVO-73-1863-s001.pdf]
